# Lactate-dependent transcriptional regulation controls mammalian eye morphogenesis

**DOI:** 10.1038/s41467-023-39672-2

**Published:** 2023-07-14

**Authors:** Nozomu Takata, Jason M. Miska, Marc A. Morgan, Priyam Patel, Leah K. Billingham, Neha Joshi, Matthew J. Schipma, Zachary J. Dumar, Nikita R. Joshi, Alexander V. Misharin, Ryan B. Embry, Luciano Fiore, Peng Gao, Lauren P. Diebold, Gregory S. McElroy, Ali Shilatifard, Navdeep S. Chandel, Guillermo Oliver

**Affiliations:** 1 Center for Vascular and Developmental Biology, Feinberg Cardiovascular and Renal Research Institute, Feinberg School of Medicine, Northwestern University, Chicago, IL 60611 USA; 2 Simpson Querrey Institute for BioNanotechnology, Northwestern University, 303 E. Superior Street, Chicago, IL 60611 USA; 3 Department of Neurological Surgery, Northwestern University Feinberg School of Medicine, Chicago, IL 60611 USA; 4 Northwestern Medicine Malnati Brain Tumor Institute of the Lurie Comprehensive Cancer Center, Feinberg School of Medicine, Northwestern University, Chicago, IL USA; 5 Simpson Querrey Institute for Epigenetics and Department of Biochemistry and Molecular Genetics, Northwestern University Feinberg School of Medicine, Chicago, IL 60611 USA; 6 Department of Oncology, St. Jude Children’s Research Hospital, Memphis, TN 38105 USA; 7 Center for Genetic Medicine, Northwestern University, Chicago, IL 60611 USA; 8 Department of Medicine, Division of Pulmonary and Critical Care Medicine, Northwestern University, Feinberg School of Medicine, Chicago, IL 60611 USA; 9 Laboratory of Nanomedicine, National Atomic Energy Commission (CNEA), Av. General Paz 1499, B1650KNA San Martín, Buenos Aires Argentina; 10 Robert H. Lurie Cancer Center Metabolomics Core, Northwestern University Feinberg School of Medicine, Chicago, IL 60611 USA; 11 Department of Ophthalmology, Duke University School of Medicine, Durham, NC 27710 USA; 12 Department of Biochemistry and Molecular Genetics, Northwestern University Feinberg School of Medicine, Chicago, IL 60611 USA

**Keywords:** Developmental biology, Organogenesis, Differentiation

## Abstract

Mammalian retinal metabolism favors aerobic glycolysis. However, the role of glycolytic metabolism in retinal morphogenesis remains unknown. We report that aerobic glycolysis is necessary for the early stages of retinal development. Taking advantage of an unbiased approach that combines the use of eye organoids and single-cell RNA sequencing, we identify specific glucose transporters and glycolytic genes in retinal progenitors. Next, we determine that the optic vesicle territory of mouse embryos displays elevated levels of glycolytic activity. At the functional level, we show that removal of Glucose transporter 1 and Lactate dehydrogenase A gene activity from developing retinal progenitors arrests eye morphogenesis. Surprisingly, we uncover that lactate-mediated upregulation of key eye-field transcription factors is controlled by the epigenetic modification of histone H3 acetylation through histone deacetylase activity. Our results identify an unexpected bioenergetic independent role of lactate as a signaling molecule necessary for mammalian eye morphogenesis.

## Abstract (web summary)

Using a combination of eye organoids and mouse models the authors identify a bioenergetic independent role of lactate as a cell signaling molecule required during early stages of eye formation in mice.

## Introduction

The vertebrate retina utilizes aerobic glycolysis (Warburg effect) to convert most of the glucose into lactate, even in the presence of oxygen, similar to actively growing mitotic tumor cells^1–6^. Non-proliferative and terminally differentiated neurons in the retina are among the most metabolically active cells in our body^6–8^. Some of the key roles of metabolism in the retina have been described in some physiological and disease contexts^9–11^. Although the vertebrate retina requires both glycolysis and oxidative phosphorylation to allow functional vision initiation and maintenance, aerobic glycolysis dominates ATP production and biosynthesis even in the growing retina during development^12–15^. However, what advantage is conferred by preferentially utilizing aerobic glycolysis in retinal tissue morphogenesis is not yet known.

Metabolic choices play a pivotal role during cell proliferation and differentiation, as metabolites support energetic demands and regulate various cellular signaling processes^4,16–20^. Although the traditional role of aerobic glycolysis is to support biomass production and ATP during rapid cell proliferation and tissue growth^4^, recent work showed that graded glycolysis along the body axis participates in embryonic tissue patterning^21,22^. Intriguingly, it was also shown that during chicken embryonic development, glycolysis-mediated regulation of intracellular pH is central to control a critical cellular signaling and posttranslational modifications such as acetylation during mesodermal differentiation^23^. Furthermore, a recent study demonstrated that lactate, the end product of glycolysis, can act as a signaling molecule that directly marks lactylation, a novel type of histone modification necessary to regulate gene expression upon macrophage polarization^24–30^. Aerobic glycolysis-derived lactate appears to play a vital role in introducing an additional regulatory layer to protein modifications and epigenetic control of gene expression in the context of immune responses, disease, and embryonic development^31,32^.

The initial morphological sign of eye formation starts with the evagination of the optic vesicles (OV)^33,34^. Similar to a variety of epithelial tissues, OV formation involves dynamic cellular movements, precise regulation of cell-cell adhesion, and cytoskeletal and extracellular matrix rearrangements^34–38^. However, the mechanisms driving genetic and epigenetic programs during epithelial morphogenesis remain unclear. We previously demonstrated that embryonic stem cell-derived eye organoids are a valuable resource to characterize the cellular and molecular steps regulating mammalian eye morphogenesis^39–41^. In the present study, we evaluated the functional role of aerobic glycolysis during retinal development. Using an approach that combines the use of eye organoids and single-cell RNA sequencing (sc-RNAseq) we initially identified an enrichment in glycolytic genes, including specific glucose transporters, in retina progenitor cell clusters during optic vesicle formation. Validating this finding, we found that glucose uptake was elevated in the anterior neural plate of mouse embryos, and conditional functional inactivation of the glucose transporter 1 (*Glut1; also known as Slc2a1*) and lactate dehydrogenase A (*Ldha*) genes displayed an impairment in eye morphogenesis. Surprisingly, addition of lactate alone to cultured eye organoids was sufficient to rescue the arrest in eye morphogenesis in cultures lacking LDH activity. Using RNA sequencing (RNA-seq) and chromatin immunoprecipitation sequencing (ChIP-seq) approaches we uncovered that lactate-mediated epigenetic modification is responsible for the upregulation of a set of key eye field transcription factors required for eye morphogenesis. These results demonstrate that lactate is a signaling molecule that plays a bioenergetics-independent role during eye morphogenesis.

## Results

### Glycolysis is required for optic vesicle morphogenesis in eye organoids

**Fig. 1:**
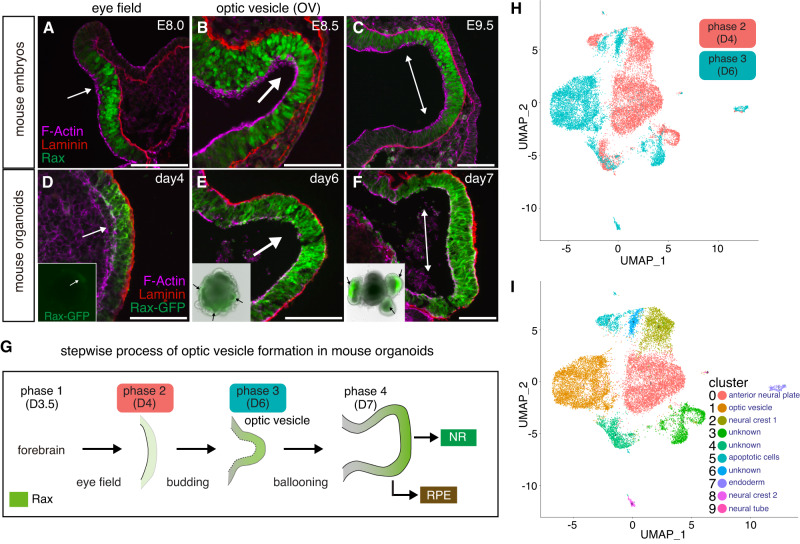
Single cell transcriptomics of mouse eye organoids during optic vesicle evagination. **A**–**F** Immunostaining of sections of optic vesicles from mouse embryos and eye organoids was performed using Laminin, Rax and GFP antibodies. DAPI and Phalloidin were used to label nuclei and F-actin, respectively. Insets show the merged images with bright fields and GFP channels at each embryonic and organoid stage. Black arrows in insets indicate evaginating OVs. Representative micrographs are shown as similar results were obtained from three independent experiments. **G** Schematic diagram showing the processes of OV evagination in eye organoids divided into four main phases. During phase 1 (day 3.5), forebrain identity is acquired; in phase 2 (day 4), weak and scattered Rax expression starts to be detected (eye field stage); in phase 3 (day 6), OV budding starts and Rax expression is increased; in phase 4 (day 7), through a ballooning process OVs fully evaginate and enlarge, and Rax expression its higher in the future neural retina territory (NR). Regions with lower levels of Rax expression will become the retina pigment epithelium (RPE). **H** Following single-cell RNA sequencing (scRNAseq) of the eye organoids, *t*-SNE plot analysis of phase 2 and phase 3 shows the obvious transcriptomic differences between these 2 stages. **I** *t*-SNE plot analysis shows each transcriptionally distinguishable cluster by unique colors. The bioinformatic analysis partitioned the cells of those two phases into 10 groups (clusters 0–9 are visualized using *t*-SNE). Each cell type is annotated based on a combination of known cell fate markers. Scale bars, 100 µm (**A**–**F**).

To better characterize the morphological and molecular steps participating in the initial process of OV and retina progenitor formation, we used eye organoids as previously described^39^. Detailed histological and immunological comparison of early stages of OV morphogenesis using mouse embryos and mouse organoids allowed us to divide this process into four main phases (Fig. 1A–G). During phase 1 forebrain identity is acquired, in phase 2 (eye field stage^33^) weak and scattered expression of *Rax*, a critical regulator of eye morphogenesis is detected, in phase 3 OV budding starts and Rax is more prominent and localized, and finally in phase 4 the OVs fully evaginate and enlarge through a ballooning process and higher Rax expression levels are seen in the future neural retina (NR), but remain lower in the prospective retina pigment epithelium (RPE). To characterize those morphological changes in further detail we performed scRNA-seq of organoids collected prior and soon after OV evagination (phase 2 and 3, Fig. 1G). Initial bioinformatic analysis confirmed that phases 2 and 3 are transcriptionally different (Fig. 1H) and include unique cluster subtypes (Fig. 1I). These cluster subtypes were annotated using a combination of known cell fate markers identified by scRNA-seq analysis as well as genes known to be required for OV formation in vivo^42^. These optic vesicle and anterior neural plate cell subtypes were validated using the available mouse embryo’s scRNAseq dataset^43,44^ (https://marionilab.cruk.cam.ac.uk/MouseGastrulation2018/).

**Fig. 2:**
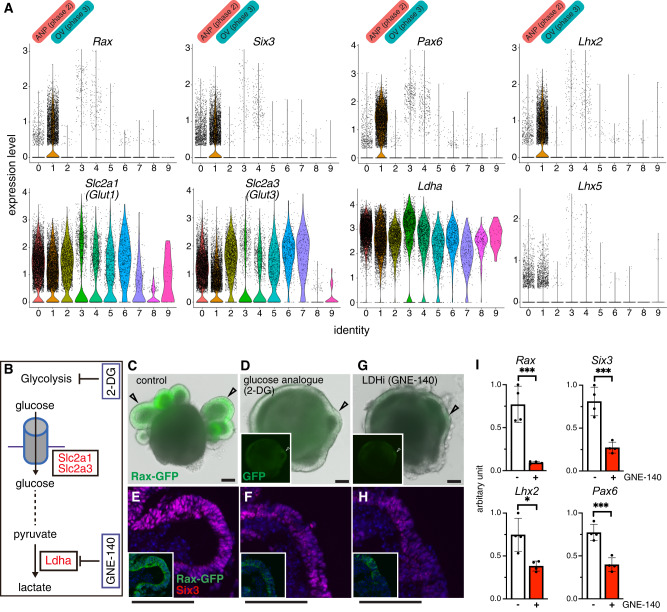
Glycolysis regulates optic vesicle morphogenesis in eye organoids. **A** Violin plots identify higher expression of the early eye differentiation markers *Six3, Rax, Pax6* and *Lhx2* on cluster 1. They also express *Slc2a1* (*Glut1*), *Slc2a3* (*Glut3*) and *Ldha*. *Lhx5* is a forebrain marker. **B**–**H** Pharmacological inhibition of glucose catabolism and LDH activity by 2-Deoxy-d-Glucose, 2-DG (2 mM) and GNE-140 (20 µM) respectively, shows that formation of RaxGFP^+^-expressing OVs (arrowheads) is arrested and the expression levels of eye markers such as Rax and Six3 is severely reduced (**E**, **F**, **H**). Insets indicate Rax-GFP expression (**D**–**H**). Representative micrographs are shown as similar results were obtained from three independent experiments (**C**–**H**). **I** Quantitative PCR analysis of a set of key eye field transcription factors known to be necessary during the process of eye morphogenesis. Scale bars, 100 µm (**C**–**H**). Unpaired Student’s *t* test (two-tailed) was performed (**I**). * or *** indicates a *p*-value is less than 0.05, or 0.001, respectively (**I**). Data are presented as mean values +/−SEM (**I**). Source data are provided as a Source Data file. *n* = 3 biologically independent experiments were performed.

It has been reported that glucose uptake in the developing tooth epithelia plays a crucial functional role in tissue morphogenesis, as blocking the activity of *Glut1/2* causes metaplasia of dental epithelial tissues^45^. In addition, high D-glucose stimulates *Pax2* gene expression and controls ureteric bud branching morphogenesis^46^. During early development, the initial glycolysis step is mediated by Glut1/3 transport of extracellular glucose inside cells^47,48^. Using UMAP and violin plot analysis of the scRNA-seq data we identified the expression of the two glucose transporter genes *Slc2a1 (Glut1)* and *Slc2a3 (Glut3)* and of the glycolytic enzyme *Ldha* in the OV and anterior neural plate subtypes (Fig. 2A). To determine whether glycolysis has any functional role during eye morphogenesis we added to the cultured eye organoids 2-deoxy-glucose (2-DG), a competitive inhibitor of glycolysis (Fig. 2B). Interestingly, 2-DG arrested OV morphogenesis, as indicated by the lack of Rax-GFP^+^-expressing OVs and by the reduction in the expression levels of typical eye markers such as Rax and Six3 (Fig. 2C–F). We next depleted the last step of glycolysis by inhibiting LDHA activity, an enzyme involved in the generation of lactate from pyruvate and the regeneration of NAD^+^^49^. Surprisingly, use of the specific chemical inhibitor GNE-140 (LDHi)^50^ to block LDH activity was sufficient to mimic the phenotype observed upon glucose inhibition, resulting in an arrest in OV morphogenesis and a reduction in the expression levels of Rax and Six3 (Fig. 2B, G, H). Quantification analysis further validated this observation, as the expression of the main eye field transcription factors was significantly reduced in the presence of GNE-140 (Fig. 2I). No significant toxicity produced by the addition of either 2-DG nor GNE-140 was observed (Supplementary Fig. 1A). Depletion of LDH activity after phase 2 did not show any major alteration in OV morphogenesis (Supplementary Fig. 1B). These results argue that glycolysis plays a critical role during OV morphogenesis in eye organoids.

### *Glut1* and *Ldha* activity are required for eye morphogenesis in mouse embryos

**Fig. 3:**
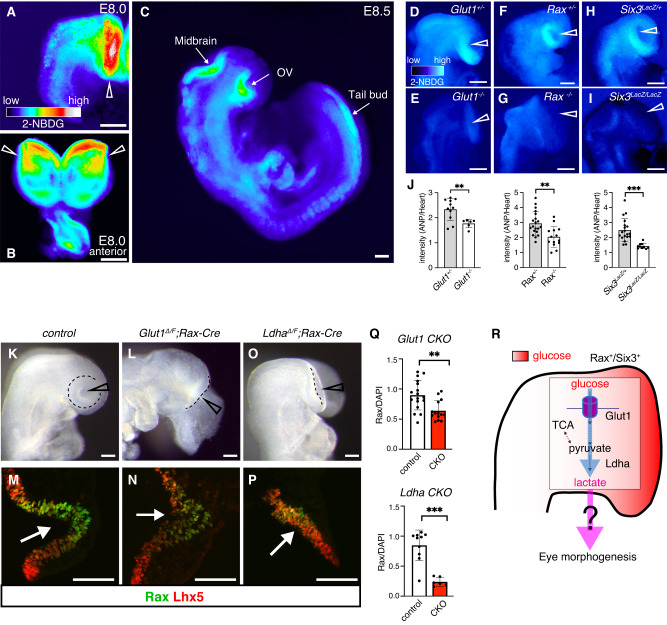
Active glucose uptake takes place in the eye field territory of mouse embryos. **A**–**C** A strong glucose uptake signal was initially detected mostly in the presumptive OVs at E8.0 (arrowheads), and in the midbrain and tail bud region at the 5-6 somite stage (E8.5). Representative micrographs are shown as similar results were obtained from three independent experiments. **D**–**I** Glucose tracer uptake was impaired in the presumptive OV territory of *Glut1*, *Rax* and *Six3* mutant embryos compared with that in their heterozygous littermates. **J** Quantification of 2-NBDG fluorescence intensity in those embryos. 2-NBDG intensity in the anterior neural plate (ANP) was measured relative to the heart primordium. **K**–**P** Bright field images and immunostaining showing that OV morphogenesis in *Glut1* and *Ldha* conditional null embryos was impaired. Rax expression was reduced, while expression of the forebrain marker Lhx5 was not dramatically affected in those mutant embryos. The Delta allele was generated by germline deletion using *Ella-Cre mice*. Only one flox allele remains upon conditional Cre expression. **Q** Quantification of Rax expression relative to DAPI signals. **R** Schematic representation of the glycolysis process during eye morphogenesis. Scale bars, 100 µm (**A**–**I**, **K**–**P**). Unpaired Student’s *t* test (two-tailed) was performed (**J**, **Q**). ** or *** indicates a p-value is less than 0.01 or 0.001, respectively (**J**, **Q**). Data are presented as mean values +/−SEM (**J**, **Q**). Source data are provided as a Source Data file. *n* = 3 biologically independent experiments were performed.

It has been reported that glycolysis regulates tail bud development in a region-specific fashion^21^. To evaluate the in vivo physiological roles of glycolysis during eye morphogenesis we first took advantage of dissected mouse embryos to monitor glucose uptake across the neural plate. To do this, we incubated wild-type (WT) embryos in eye organoid’s medium (RDM) containing the fluorescent tracer 2-NBDG. Intriguingly, specific strong uptake signal was detected mostly in the presumptive OVs territory within the anterior neural plate (Fig. 3A, B). Half a day later, while the signal in the OVs remained, it was also evident in the midbrain and tail bud (Fig. 3C). Consistent with this observation, Glut1 and Glut3 are reported to be expressed in the presumptive OV territory, although Glut1 expression is higher than that of Glut3^51^. It was previously described that *Glut1* null embryos have severe growth retardation and major morphological abnormalities, including the lack of visible eyes^52^. To functionally validate the activity of the glucose transporter in vivo, we took advantage of available *Glut1* mutant mice to evaluate whether this gene is responsible for the observed glucose uptake. As shown in Fig. 3E, glucose tracer uptake was impaired in the presumptive OV territory of these mutant embryos when compared with that in their heterozygous littermates (Fig. 3D). This result suggests that Glut1 likely mediates glucose uptake in the developing eye territory. This outcome led us to question whether the eye field fate or lineage has a causal relationship with the observed glucose uptake impairment. To test this possibility, we took advantage of *Rax* and *Six3* null embryos whose rostral neural identity, including the eye field is completely missing^53,54^. Surprisingly, we also observed a substantial reduction of the glucose tracer signals in the rostral most part of *Rax* and *Six3* mutant embryos (Fig. 3F–J). These results suggest that Rax and Six3-promoted eye identity is required for tissue-specific glucose uptake during mouse eye formation.

To conclusively evaluate whether *Glut1* activity is physiologically required during eye morphogenesis, we generated conditional null embryos using the eye field specific *Rax-Cre* mouse strain^55^. Interestingly, *Glut1* conditional mutant embryos showed impaired eye structures at E8.5 (Fig. 3K, L). To characterize the mutant phenotype in further detail, we performed immunostaining using Rax and the forebrain marker Lhx5^56^. Supporting the result discussed above, in mutant embryos Rax expression was reduced (Rax activity is critical to induce the eye field territory and OV evagination^53^), whereas that of Lhx5 appears normal (Fig. 3M, N). It was recently shown that lactate production is correlated with lung branching morphogenesis, as Ldha displayed specific expression in a region where the distal lung buds progressively grow^57^. Homozygous null *Ldha* embryos are early lethal after implantation, probably because impairing glycolysis results in the loss of the main source of ATP^58^. Because of this early lethality, to impair lactate production in the eye territory we generated *Ldha;Rax-Cre* conditional null embryos. Similar to *Glut1* conditional null embryos, we found that OV formation is arrested and Rax expression is severely reduced in these mutant embryos (Fig. 3O, P). Quantification analysis performed in *Glut1;Rax-Cre* and *Ldha;Rax-Cre* conditional null embryos corroborated the significant reduction in the expression of the eye developmental marker Rax (Fig. 3Q). This reduction and the arrest in OV formation in *Glut1* mutant embryos were not as severe as in *Ldha* mutant embryos (Fig. 3L–Q). We cannot rule out the possibility that other glucose transporters in the OVs could play a compensatory role and partially restore OV morphogenesis. These results revealed that glycolysis is essential for eye morphogenesis in vivo (Fig. 3R).

### Fate tracing of glucose identified elevated lactate levels in eye organoids

**Fig. 4:**
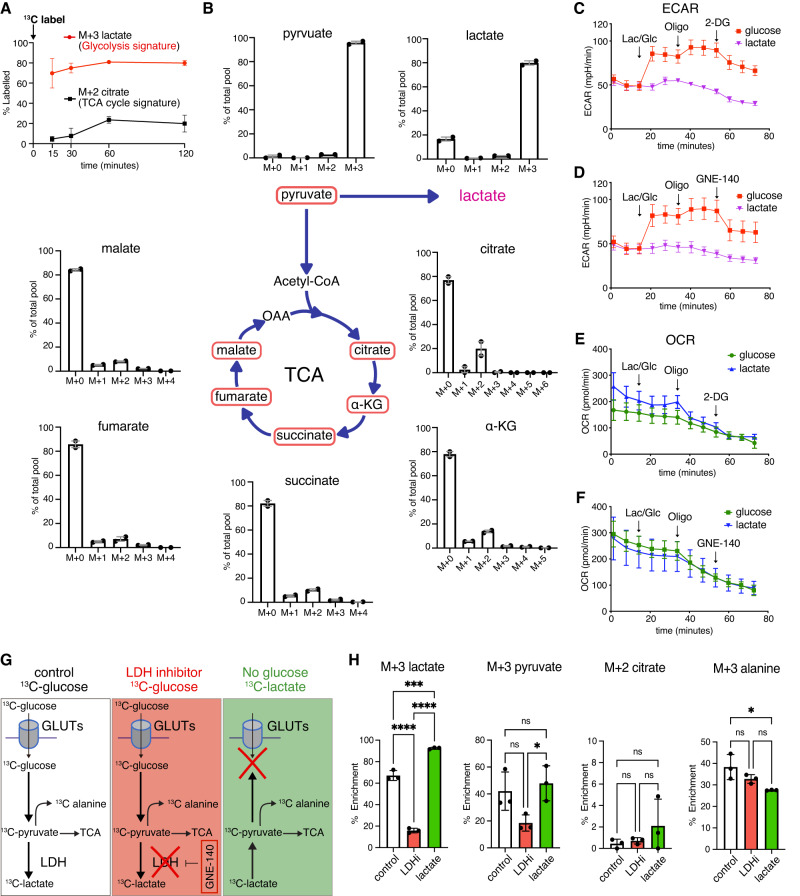
^13^C-isotope labeling reveals the profile of glycolytic intermediates during eye morphogenesis. **A**, **B** The direct quantification of glucose consumption using ^13^C glucose time-course tracing was performed in cultured eye organoids and measured by LC-MS/MS analysis. Amongst all detected glycolytic intermediates, the main ^13^C glucose-derivatives were pyruvate and lactate, while the levels of citrate, one of the signature metabolites of the TCA cycle were extremely low. The M + 0, M + 1, M + 2 and M + 3 metabolites were measured as % of the total pool to distinguish unlabeled and labeled metabolites. The labeled lactate during glycolysis saturated up to 80%. In the TCA cycle, citrate shows that only about 20% of the succinate pool was ^13^C labeled. **C**–**F** To evaluate glycolysis and mitochondrial respiration, seahorse analysis was performed to measure eye organoids-derived extracellular acidification rate (ECAR) and oxygen consumption rate (OCR). Injecting glucose (Glu) elevated ECAR whereas lactate (Lac) did not. Oligomycin (Oligo), an inhibitor of ATP synthase necessary for oxidative phosphorylation further increased ECAR levels. Subsequent addition of either 2-DG or GNE-140 depleted the ECAR to the basal level. **G**, **H** ^13^C-glucose labeling under three conditions: ^13^C-glucose = control (white scheme), ^13^C-glucose + LDHi = LHDi (red scheme), ^13^C-lactate in glucose free media mimicking the lack of glucose uptake = lactate (green scheme). Treatment with LDHi (which prevents OV morphogenesis) significantly reduced ^13^C-labeled lactate; however, it did not change the levels of pyruvate, citrate and alanine. ^13^C-lactate was not dramatically metabolized into TCA cycle specific metabolites as seen in M + 2 citrate. One-way ANOVA followed by Tukey’s post-hoc test was performed (**H**). ****p < 0.0001, ***p < 0.001, *p < 0.05, n.s. not significant (**H**). Data are presented as mean values +/−SEM (**H**). Source data are provided as a Source Data file. *n* = 3 biologically independent experiments were performed.

To characterize glycolysis-derived metabolites that might participate in eye morphogenesis we used a glucose fate-tracing approach. Glucose is catabolized by glycolysis to pyruvate that can be converted into alanine, lactate or enter mitochondria^59^. Glucose can also enter multiple branched pathways from glycolysis including the hexosamine, pentose phosphate, and de novo serine biosynthetic pathways^59^. To identify the endogenous biochemical cascade in OV progenitor cells, we performed a direct quantification of glucose consumption using ^13^C-glucose tracing experiments followed by liquid chromatography–mass spectrometry (LC-MS/MS) analysis to profile downstream metabolites and their dynamics inside a cell^60^. A major analytical challenge with this procedure is the amount of material that can be obtained per embryo, and the certainty that they are all at the correct developmental stage. To avoid this problem, we took advantage of the eye organoids to pool 50 organoids per time point and/or condition. Because the natural abundance of isotopic metabolites in the early eye organoids has not yet been reported, we have initially performed LC-MS/MS without exogenous isotopic labeling to show the natural abundance of isotopic carbons. The natural abundance of M + 1 isotopologues was less than 5% for every metabolite measured, whereas for the M + 2/4 TCA cycle and other glycolytic intermediates it was essentially 0. For any higher-order labeling more than M + 2, natural abundance was undetectable in most metabolites (Supplementary Fig. 2A, B). The exception was the natural abundance of M + 3 labeled lactate that is approximately 4% (the average 3.97 SEM +/−2.00%, Supplementary Fig. 2B). Next, eye organoids were cultured in medium containing uniformly labeled ^13^C-glucose, and the label propagation in glycolytic metabolites was measured at several time points within the first 120 min after the addition of ^13^C glucose. Amongst all detected glycolytic intermediates, the majority of ^13^C glucose-derivatives was lactate, while citrate, one of the signature metabolites of the TCA cycle exhibited extremely low levels over the entire labeling measurement (Fig. 4A). Intriguingly, half of the glycolytic lactate became labeled in the first 15 min and saturated up to 80% (Fig. 4A). By contrast, citrate in the TCA cycle took 1 h to reach steady state, wherein only about 20% of the citrate pool was ^13^C labeled (Fig. 4A). As shown in Fig. 4B, labeling was reduced in TCA cycle metabolites as the cycle progressed, while pyruvate production reached more than 90% (Supplementary Fig. 3A). This initial isotopic labeling analysis indicates less activity of the TCA cycle in the eye organoids. It is also important to collect all the intermediate metabolites over the given period during the time-course analysis, as the TCA cycle undergoes multiple rounds^61,62^. Thus, we also measured M4, M5, M6 of TCA cycle metabolites and pyruvate carboxylase mediated M1 and M3 labeled metabolites. However, as the labeling through the TCA cycle is limited in these organoids (Fig. 4B and Supplementary Fig. 3B), we observed little to no labeling of M + 4 intermediates, indicating that over two hours only one turn of the TCA cycle occurred. Regarding other intermediates, we found no detectable M + 1,3,5 labeling indicating that pyruvate carboxylase activity in these organoids was negligible. Critically, unlike LDH inhibition, functional inhibition of the mitochondrial pyruvate transporter (MPC) by UK5099^63^ did not prevent *Rax* expression (Supplementary Fig. 3C, D).

To further evaluate glycolysis and mitochondrial respiration, Seahorse analysis was performed to measure eye organoids-derived extracellular acidification rate (ECAR) and oxygen consumption rate (OCR). We observed that as expected, glucose administration increased ECAR which was further increased with oligomycin treatment that blocks the mitochondria electron transport process (Fig. 4C, D). Treatment with either 2-DG or LDHi significantly reduced ECAR (Fig. 4C, D). It is worth noting that addition of lactate, the final glycolysis product, did not show any noticeable change of ECAR, which explains the dynamic change in acidity originated mainly from glycolysis through the initial metabolite source, glucose. By striking contrast, OCR did not show any significant difference between glucose and lactate addition (Fig. 4E, F). Furthermore, the addition of either glucose or lactate had no influence on OCR, indicating a minimal use of these substrates for TCA metabolism. This metabolite profiling and the results from the functional ablation of MPC strongly suggest that glucose in OV progenitors is preferentially metabolized into lactate rather than into TCA metabolism. Collectively, it is possible that glucose-derived lactate functionally participates in eye developmental programs.

As stated above, glucose can be fated into multiple branched signaling pathways. To examine the potential involvement of all the metabolites detected in the initial time-course tracing analysis, we performed ^13^C-glucose labeling under three conditions: ^13^C-glucose medium (control), ^13^C-glucose + LDHi medium (LDHi) and ^13^C-lactate in glucose-free medium (lactate) (Fig. 4G). Because the activity of LDHA is required for lactate production, the addition of its inhibitor significantly reduced M + 3 lactate (Fig. 4H). As expected, lactate was detected by the exogenously added ^13^C-lactate in the absence of exogenous glucose (Fig. 4H). On one hand, the TCA metabolites citrate and succinate did not change significantly by blocking LDH activity (Fig. 4H and Supplementary Fig. 4A). The result agrees with the proposal that TCA metabolites are unlikely to participate in the active process of OV morphogenesis. However, another possibility could be that alanine, which is further metabolized from pyruvate and was ^13^C-labeleled in our assay (Supplementary Fig. 3E), also participates in the process of eye morphogenesis^64^. However, this alternative was not validated by our assay showing that depletion of LDH activity did not cause any reduction in ^13^C-labeleled alanine (Fig. 4H). Importantly, LDH depletion or ^13^C-lactate addition also failed to reduce or rescue M + 3 glycerate labeling, respectively (Supplementary Figs. 3E and 4B). Lastly, LDH inhibition had no effect on nucleotide synthesis and ^13^C-lactate labeling failed to rescue nucleotide labeling in the absence of glucose (Supplementary Fig. 4C), suggesting that OV morphogenesis is independent of the pentose-phosphate pathway. In summary, this analysis demonstrates that lactate likely plays a direct role in OV morphogenesis.

### Lactate mediates eye morphogenesis

**Fig. 5:**
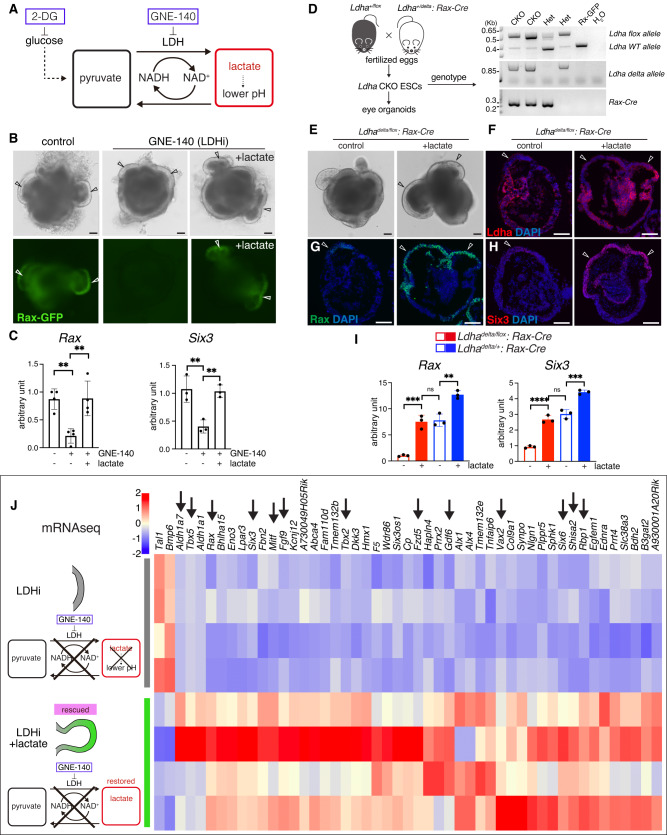
Lactate is required for eye morphogenesis by regulating eye transcriptional programs. **A**, **B** The addition of sodium L-lactate, which does not change the pH, significantly rescued the OV phenotype (arrowheads) in the presence of 20 µM GNE-140 (LDHi). **C** Quantification analysis using RT-qPCR confirmed that *Rax* and *Six3* expression were also rescued by the addition of sodium L-lactate (25 mM). **D** Generation and genotyping of *Ldha*^*delta/flox*^*;Rax-Cre* conditional mouse embryos (CKO) and derivation of ESCs. The delta allele was generated by germline deletion using *Ella-Cre* mice. Only one flox allele remains upon conditional Cre expression. Representative micrographs are shown as similar results were obtained from three independent experiments. **E** Genetic deletion of LDHA in the eye field-specific region leads to defects in optic vesicle formation in *Ldha* mutant organoids. The addition of lactate rescued the eye defects as indicated by the formation of OVs (arrowheads). **F**–**I** Immunostaining and RT-qPCR analyses showed that *Ldha*-depleted organoids recovered Rax and Six3 expression in the presence of 25 mM lactate (arrowheads); however, Ldha expression in the mutant OVs is not detected (**F**). **J** RNA-sequencing was performed to compare LDH block (LDHi) vs LDHi plus sodium L-lactate. Addition of lactate resulted in significant transcriptional changes as indicated by heatmap analysis that identified changes in the expression of various genes known to regulate eye formation (arrows). Scale bars, 100 µm (**B**, **E**–**H**). One-way ANOVA followed by Tukey’s post-hoc test was performed (**C**, **I**). ****p < 0.0001, ***p < 0.001, **p < 0.01, n.s. not significant (**C**, **I**). Data are presented as mean values +/−SEM (**C**, **I**). Source data are provided as a Source Data file. *n* = 3 biologically independent experiments were performed.

LDH activity has multiple roles in the glucose pathway, including the production of lactate that can be converted back into pyruvate^65^. As seen in Fig. 4H, isotopic labeling analysis showed that pyruvate was converted from exogenous ^13^C lactate. Accordingly, we evaluated whether pyruvate could rescue the loss of OV morphogenesis caused by the addition of the LDH chemical inhibitor GNE-140 (Supplementary Fig. 5A). To facilitate pyruvate incorporation inside the cells, we added a membrane-permeable form of pyruvate^66^ to the eye organoids in the presence of GNE-140. However, no noticeable rescue was observed by the addition of methyl-pyruvate (Supplementary Fig. 5B, C). It has been reported that glycolysis-produced lactate plays a pivotal role in pH modulation during mesodermal differentiation in tail bud development^23^. To test whether pH controls eye morphogenesis, we used media that changes pH in a graded fashion in the presence of the LDH inhibitor. Intracellular pH (pHi) responds to changes in extracellular pH (pHe) when a cell re-balances transmembrane acid–base fluxes that alters steady-state pHi^67^. Unlike the mechanism in tail bud development, lowering the pH did not show any noticeable change in OV morphogenesis and did not rescue *Rax* expression levels (Supplementary Fig. 5D, E). Next, we tested if lactate itself could rescue the eye defects. Surprisingly, addition of sodium L-lactate, which does not change the pH, significantly rescued the defect in eye morphogenesis in the presence of either 2-Deoxy-D-glucose or GNE-140 (Fig. 5A, B and Supplementary Fig. 5F, G). Quantification analysis using qPCR confirmed that *Rax* and *Six3* expression is also rescued by the addition of lactate (Fig. 5C). To further validate this important observation in vivo we took advantage of our organoid system^41^ to genetically delete *Ldha* in the anterior neural plate. *Ldha*^*flox/delta*^*;Rax-Cre* blastocysts were used to isolate ESCs for the eye organoid differentiation assay (Fig. 5D and Supplementary Fig. 6A). Initially, day 3 *Ldha* CKO organoids expressed Ldha in the surrounding neural ring (Supplementary Fig. 6B). By day 7, *Ldha* CKO organoids exhibited successful deletion of LDHA expression in Rax-expressing cells; LDHA expression remained in heterozygous organoids (Supplementary Fig. 6C). As expected, in the mutant organoids expression of the developmental eye markers *Rax* and *Six3* was reduced significantly, while that of the pan-neural marker *Sox2* was not affected (Supplementary Fig. 6D). Next, we found that lactate addition was sufficient to rescue the OV defects in the CKO organoids (Fig. 5D–F) and as expected, expression of typical eye developmental markers was elevated (Fig. 5G–I).

### Lactate drives transcriptional programs for tissue morphogenesis

Lactate production can regulate a wide variety of biological processes, including angiogenesis, hypoxia, and immune cell function^68–70^. However, little is known about its possible transcriptional impact on signaling pathways regulating tissue morphogenesis. To identify lactate-driven transcriptomic regulation we performed RNA-seq comparing control organoids vs LDHi treated organoids and LDHi treated vs LDHi treated plus lactate (condition that rescues the OV defect). To identify lactate-dependent transcriptional changes, we initially performed a direct comparison with (LDHi+lactate) or without (LDHi) lactate. Surprisingly, heatmap representation revealed that addition of lactate resulted in significant transcriptional changes, including that of various genes known to be necessary for eye morphogenesis (Fig. 5J). Intriguingly, the eye field transcriptional factors *Six3*^71^ and *Rax*^53^ were both upregulated by lactate concomitant with the rescue in eye formation. Furthermore, we identified upregulation of additional genes known to regulate eye morphogenesis such as *Fzd5, Shisa2, Tbx2* and *Pax2* whose expression changed dramatically upon functional depletion of Ldha and lactate addition (Fig. 5J). As shown previously, *Fzd5* mutant mice failed to complete optic cup morphogenesis^72^. *Shisa2* loss of function or overexpression in *Xenopus* induces small eyes or ectopic eyes, respectively^73^. Loss of *Tbx2* causes a delay in OV morphogenesis leading to small and abnormally shaped optic cups^74^. *Pax2* mutations and loss of function are associated with coloboma or microphthalmia, both of which are consequences of eye morphogenesis defects^75,76^. Changes in the expression of various other genes known to participate in different steps of eye morphogenesis are indicated by arrows in the heatmap (e.g., *Mitf, Fgf9, Aldh1a7, Gdf6, Rbp1, Tbx5, Six6* and *Vax2*) (Fig. 5J)^77–84^. These findings allow us to conclude that glycolysis-derived lactate plays a central functional role in bridging a genetic program with eye morphogenesis.

### Global profiling of epigenetic modifications by glycolysis-derived lactate

**Fig. 6:**
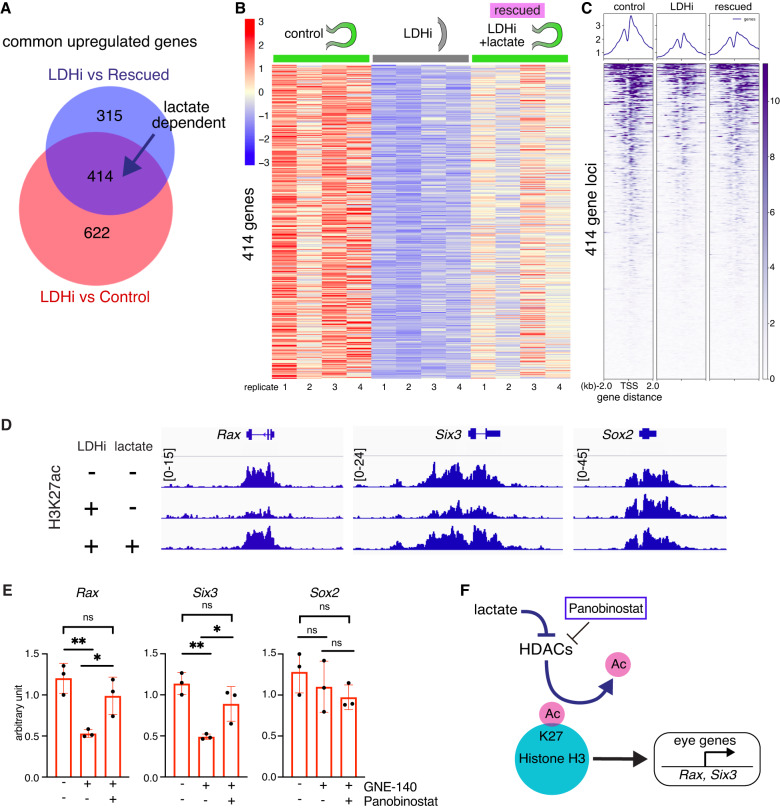
HDAC and CBP/p300 activity specifically regulate eye developmental genes. **A** Venn diagram reveals genes whose expression is dependent on lactate. **B** Heatmap of 414 lactate dependent differentially expressed genes. **C** Global histone H3K27 acetylation (H3K27ac) chromatin immunoprecipitation sequencing (ChIPseq) was performed in the following two conditions: 1) addition of the LDH inhibitor (20 µM), 2) addition of the LDH inhibitor in the presence of lactate (25 mM). The ChIP occupancy heatmaps showing H3K27ac plots at transcription start sites (TSS) (Enhancers/Promoters, +/−2 kb) of the differentially expressed genes identified from the RNA-seq analysis. **D** Bioinformatic analysis was performed and the ChIPseq data was visualized using the free software IGV (version 2.4.14) around coding and regulatory regions such as putative promoters and/or enhancers of some typical eye gene loci (*Rax*, *Six3*). Relatively higher peaks are seen in comparison with those upon adding the LDH inhibitor. This peak reduction when adding LDHi was recovered upon lactate treatment. **E** The pan-HDAC inhibitor Panobinostat was added to the organoids in the presence of LDHi from day 4 to day 5. Dual inhibition by LDHi and HDACi rescued eye marker genes at day 5, whereas pan-neural gene, *Sox2* did not change dramatically as seen by qPCR. **F** Potential mechanisms regulating the eye developmental program through H3K27ac involving histone acetyltransferases and deacetylases. Panobinostat, a potent inhibitor depletes histone deacetylases (HDACs) mediated H3K27-deacetylation process. One-way ANOVA followed by Tukey’s post-hoc test was performed (**E**). **p < 0.01, *p < 0.05, n.s. nonsignificant (**E**). Data are presented as mean values +/−SEM (**E**). Source data are provided as a Source Data file. *n* = 3 biologically independent experiments were performed.

Metabolites regulate epigenetic modifications to control gene expression and cell differentiation^85,86^. Histone acetylation is generally associated with active transcription^87^. However, whether glycolysis-derived metabolites mediate epigenetic modifications important during organ morphogenesis in general, or eye development in particular has not yet been shown. To determine whether lactate participates in epigenetic regulation required for eye morphogenesis, we performed global ChIP-seq across the genome. It is widely known that lysine 27 acetylation of histone H3 (H3K27ac) is a histone marker enriched at promoters and enhancers. Accordingly, we used a well-characterized antibody recognizing H3K27ac in chromatin isolated from eye organoids cultured with and without addition of the LDHA inhibitor. Bioinformatic analysis identified differential peaks close to gene regulatory regions. To facilitate narrowing the putative candidate genes we cross-compared the ChIP-seq and identified 414 lactate dependent genes from our RNA-seq (Fig. 6A–C). The ChIP-seq occupancy analysis determined that whereas overall genome wide H3K27ac patterns were unaffected, there was a specific loss of H3K27ac at key eye morphogenesis genes in response to LDHi (Fig. 6C, D and Supplementary Fig. 7A, B). This striking result implicates lactate as a specific key regulator involved in H3K27ac at eye developmental loci.

**Fig. 7:**
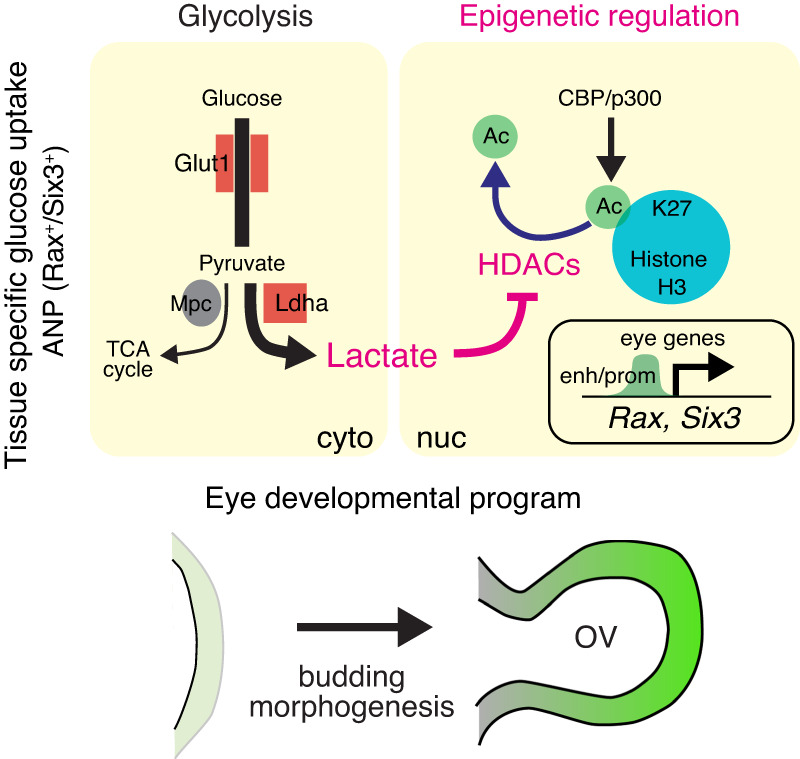
Bioenergetics-independent role of lactate during eye morphogenesis. Glycolysis-derived lactate possesses non-metabolic functions that contribute to transcriptional regulation through epigenetic pathways involving histone H3 acetylation writer and eraser. Higher glucose uptake begins in the anterior neural plate (ANP) prior to optic vesicle (OV) morphogenesis. While the activity of *Glut1* and *Ldha* is required for canonical glycolysis, the final product of glycolysis, lactate, acts as a signaling molecule being likely an endogenous HDAC inhibitor during eye morphogenesis. This non-canonical regulation is ATP-production independent and pivotal for specific eye developmental programs. cyto cytoplasm, nuc nucleus, enh enhancers, prom promoters.

To explore the direct role of lactate in the eye developmental program, we sought to find potential links between lactate and histone epigenetic modifiers, such as the Histone H3 acetylation writers and erasers whose specific epigenetic activities are pivotal for normal development and disease^88–91^. The writers, CBP and p300 act as transcription coactivators by interacting with a variety of specific transcription factors and co-regulators^92^. Standard null p300 mutant embryos show severe head defects with small eyes^93^. CBP and p300 are required for rod and cone photoreceptor neurons and lens induction^94–96^, suggesting CBP/p300-mediated cell fate control is important for eye development. By contrast, for one of the erasers, *Hdac1*-null embryos displayed numerous defects including severe head abnormalities leading to embryonic lethality before E9.5^97^. *Hdac1* and *Hdac2* are implicated in early neural development as *Nestin-Cre* mediated conditional deletion of those two genes caused severe brain defect^98^. *Hdac3* null embryos die before E9.5 due to defects in gastrulation^99^. Accordingly, HDACs play important roles in early embryogenesis, including brain morphogenesis. Interestingly, a previous report demonstrated that lactate is an endogenous inhibitor of histone deacetylase activity^100^. To seek the link between lactate and HDACs, we performed gene expression analysis of eye organoids and observed that LDHi mediated downregulation of key eye developmental genes, such as *Rax* and *Six3*, was rescued by the chemical inhibitor Panobinostat, a compound known to inhibit HDAC activity^101,102^ (Fig. 6E, F). Notably, this effect is specific to eye genes as the pan-neural marker *Sox2* was unaffected (Fig. 6E). This result provides a direct link between histone acetylation and the eye developmental program. To further dissect this link, we used a well-characterized CBP/p300 acetyltransferase inhibitor (A-485). The addition of A-485 resulted in specific downregulation of eye-determining factors (Supplementary Fig. 8A, B). The result identified the histone acetylation writer CBP/p300 as a crucial factor involved in the specific regulation of eye development. These lines of evidence suggest that the role of lactate could be to inhibit HDAC enzymatic activity, presenting a direct link between histone acetylation and the eye developmental program. Altogether, we propose that glycolysis-produced lactate in the anterior neural plate controls eye morphogenesis at the epigenetic level through histone deacetylase (Hdac) activity (Fig. 7).

## Discussion

Using a combination of eye organoids and mouse genetics we now report that: a) scRNA-seq from eye organoids identified key glycolytic genes expressed in developing OVs; b) the eye field territory of mouse embryos displays elevated glycolytic activity; c) lactate production is required for OV morphogenesis; d) glycolysis-mediated lactate upregulates eye field transcription factors and associated epigenetic modifications (H3K27ac). Together, these results identified an unexpected functional role of aerobic glycolysis during mammalian organogenesis (Fig. 7). Our data argue that lactate acts as a signaling molecule playing diverse physiological roles during organ morphogenesis.

Metabolic signals dictate cell fate and cell morphology by contributing to transcriptional programming, epigenetic modifications, and biochemical differentiation cues^103^. For example, glycolysis is important for physiological wound healing in the skin through upregulation of Glut1 and related glycolytic enzymes^104^. The RNA-binding protein Lin28-mediated translation of gene products in glycolysis facilitates skin tissue repair by reprogramming cellular bioenergetics^105^. In the adult stage, LDH and lactate are well-known biomarkers of a variety of tissue injuries, also presenting critical functional activity, for instance for neural regeneration post-spinal cord injury^65,106–109^. These few reports suggest that glycolysis might play a variety of roles in tissue repair and wound healing processes. Our metabolomic and transcriptomic analysis indicate that there is a crosstalk between metabolism and tissue morphogenetic programs, leading to the possibility that metabolic regulation of cellular signaling is reused in tissue repair or remodeling processes. Future investigations may focus on glycolysis-mediated tissue repair and regeneration in a variety of embryonic and adult cell types.

In relation to lactate-mediated regulation of protein acetylation, it has been shown that glycolysis creates favorable chemical conditions for acetylation of the signaling molecule β-catenin that is important for tail bud development^23^. This protein acetylation mechanism is likely conserved across vertebrate developmental programs including humans. Conversely, protein deacetylation in the nucleus is regulated by HDAC family members, which target a variety of histones, particularly histone H3. Our results demonstrate that lactate, a putative HDAC inhibitor, regulates local histone acetylation associated with the specific eye developmental program. These findings raise several exciting future directions for the study of metabolites-dependent regulation of HDAC activity in eye morphogenesis.

It was recently reported that endogenous elevation of lactate to ~15 mM preceded the timed degradation of cyclin B1 and securin, and mitotic exit^110^. Accumulation of intracellular lactate prior to mitosis coincided with stable expression of major enzymes that regulate pyruvate utilization^110^, and importantly no detectable proteome-wide evidence for protein lactylation was observed^110^. All these data support the argument that lactate may not work only through protein lacylation whether it is histone or any other target. It is likely that lactate could have multiple effects such as HDAC inhibition, maybe context-dependent lactylation of proteins including histones or inhibition of the SENP1 protease. In our model, lactylation is unlikely a major player in OV morphogenesis. However, we cannot exclude the possibility that lactate produced in OVs may increase histone lactylation.

As the last step of glycolysis, the enzymatic activity of LDHA reduces pyruvate into lactate and regenerates NAD^+^ from NADH^49^. The regeneration of cytosolic NAD^+^ is required for sustaining cytosolic glucose catabolism by glycolysis^58,111^. In our RNAseq analysis we identified 414 common genes regulated by LDH and lactate (Fig. 6A). However, the 622 genes listed when comparing control vs LDHi-treated could certainly be consequence of changes in the NAD^+^/ NADH ratio. We demonstrated that addition of lactate is sufficient to rescue the process of eye morphogenesis arrested by chemical and genetic ablation of LDH activity (Fig. 5B, E), a result suggesting that NAD^+^ is unlikely the major player in this process. In this study, we were able to dissect the critical glycolytic process regulated by lactate-mediated transcriptional mechanisms independent from its well-recognized bioenergetic roles.

In summary, glycolysis-derived lactate possesses non-metabolic functions that contribute to transcriptional regulation through epigenetic pathways involving histone H3 acetylation writers and erasers. Higher glucose uptake begins in the anterior neural plate prior to OV morphogenesis. While the activity of *Glut1* and *LdhA* is required for canonical glycolysis, the final product of glycolysis, lactate, acts likely as an endogenous HDAC inhibitor during eye morphogenesis. This non-canonical regulation is ATP-production independent and pivotal for specific eye developmental programs.

## Methods

### Ethics statement

Our research complies with all relevant ethical regulations and work with mice has been approved by Northwestern University Institutional Animal Care and Use Committee Office.

### Optic vesicle differentiation from embryonic stem cells

Mouse embryonic stem cell Rax-EGFP was obtained originally from Dr. Yoshiki Sasai and now is deposited under the name AES0145: Rx-GFP K/I EB5 at RIKEN cell bank. *Rax-GFP* ESCs were maintained on gelatin-coated dishes in ESC maintenance medium with 2000 U/mL leukemia inhibitory factor (LIF) and 20 μg/mL blasticidin as described previously^39^. Cells were passaged every 2–3 days. For differentiation, a standard eye organoid protocol was used^112^. Briefly, 3000 cells per 100 μL Glasgow minimum essential medium (GMEM) containing 1.5% knockout serum replacement (KSR) (Life Technologies; called retinal-differentiation medium [RDM]^112^; per well were plated in Nunclon Sphera 96-Well, Nunclon Sphera-Treated, U-Shaped-Bottom Microplate (ThermoFisher Scientific, 174929 or 174925) on day 0. On day 1, Matrigel Matrix growth factor reduced (final concentration, 200 μg/mL) (Corning, 354230) in RDM was added to the aggregates, which were maintained in culture until day 7. Unless stated otherwise, for the pharmacological experiments, 2-Deoxy-D-glucose (2 mM, SIGMA), GNE-140 (20 µM, SIGMA or MedChemExpress) or UK5099 (1 µM, MedChemExpress) were added and incubated for 3 days and imaged (EVOS, Cell Imaging System, ThermoFisher), immunostained and analyzed by RT-qPCR (QuantStudio 3, ThermoFisher Scientific). For the rescue experiments, graded methyl-pyruvate (SIGMA)^66^, sodium-L-lactate (25 mM, SIGMA) or eye media with a graded pH (7.8, 7.4, 7.0, 6.6) were added to the eye organoid culture in the presence of 2-Deoxy-D-glucose or GNE-140 for 3 days. The graded pH was prepared prior to the rescue experiment to evaluate the effect of pH in cultured eye organoids. The rationale for this pH range is that it has been shown that extracellular pH (pHe) of ~6.6 to~8 is an appropriate physiological range that a cell would encounter in-vivo, and that range is most commonly tested^113–117^). Supporting the pH range used in this assay, a recent review exploring the range of pH in normal and tumor tissues found that pH is in the same range that we tested in our experiments^117^. Therefore, the range of pH is consistent with the previous literature and close to the physiologic situation in tissues. The samples were collected for imaging (EVOS), immunostaining and analyzed by RT-qPCR. Panobinostat (0.05 µM, Selleck Chemicals) and A-485 (2 µM, MedChemExpress) were added to the culture at differentiation day 4 for a day, followed by gene expression analysis. Embryonic stem cell–qualified fetal bovine serum, KSR and Matrigel Matrix were used for the eye organoid culture.

### Single cell RNA sequencing

For preparation of single cells, the eye organoids were collected into 15 mL tubes. Then the tissue was washed with PBS twice and 0.75 mL of warmed Papain (Worthington Biochemical Corporation) was added with DNase I (1:1000, Sigma) and Y-27632 (10 µM, Millipore) and incubated at 37’C. After 45 min, the tissue was carefully pipetted, and a stop solution was added. The dissociated solution was filtered with a 100 µm cell strainer (Falcon; Corning) and centrifuged at 1000 rpm at room temperature (RT) for 5 min. 1 mL HBSS (0.2% BSA fraction V + 10 µM Y-27632) was added to the cell pellets and then filtered using a 40 µm cell strainer (Falcon; Corning). Cells were counted using a Cellometer K2 (Nexcelom) with acridine orange to calculate total number of nucleated cells and propidium iodide to count dead cells (cell viability was 89%). The library preparation and sequence were performed as described previously^118^. The single cell organoid samples from three biologically independent experiments were pooled prior to the library generation. Single-cell 3′ RNA sequencing libraries were prepared using Chromium Single Cell v2 Reagent Kit and Controller (10X Genomics, Pleasanton, CA, USA). Libraries were assessed for quality (TapeStation 4200; Agilent, Santa Clara, CA, USA) and then sequenced on an HiSeq 4000 instrument (Illumina, San Diego, CA, USA). Raw reads were demultiplexed, filtered, and mapped to the mouse reference genome (mm10) using Cell Ranger 7.0.0 with default settings. The raw feature-barcode matrices generated by Cell Ranger were used for downstream analysis using Seurat v4.1.1. To exclude low-quality cells, standard filters were applied (cells with fewer than 500 genes and higher than 5000 genes) (cells with fewer than 500 UMIs (unique molecular identifiers) and over the 90th quantile of total UMIs (26000) (cells with more than 10% mitochondrial genes) (cells with fewer than 20% ribosomal genes) (cells with higher than 0.5% hemoglobin genes). The cell counts for phase 2 was 9949 cells and for phase 3 7977 cells. The filtered expression data was normalized for each cell by the total expression, multiplied by a scale factor of 10,000, and then log-transformed. The cell-cycle phase scores were calculated for regression. A total of 12792 variable genes were identified. The data was scaled using ScaleData (cell cycle results S.Score and G2M.Score, percentage of mitochondrial genes, number of features, and sample identities were regressed) to remove batch effects. Dimensionality reduction was done using Principal Component Analysis (PCA) and based on the results 50 PCs were used to identify clusters. The clusters were identified using the Approximate Nearest Neighbors method at different resolutions. After analyzing the different resolutions, 0.2 was chosen for further analysis and 10 clusters were identified. Marker genes were identified using FindAllMarkers (MAST method).

### Generation of mutant mouse strains

*Six3*^*LacZ*^ mice were previously described^54^. Genotyping was performed using specific primers to identify the WT and *LacZ* null alleles. *Rax* flox mice were kindly provided by Dr. Seth Blackshaw (Johns Hopkins University) and were previously described^119^. *Rax* flox mice were crossed with *E2a-Cre*^120^(Jackson lab #003724) kindly provided by Dr. Susan Quaggin (Northwestern University). The F1 animals were genotyped with specific primers to detect germline deletion to obtain the *Rax* KO allele^119^. *Ldha* flox mice were purchased from Jackson lab (Strain #030112). *Ldha* flox mice were crossed with *E2a-Cre* and F1 animals were genotyped using specific primers to identify the germline delta allele. *Glut1* flox mice were kindly provided by Dr. E. Dale Abel (University of Iowa) and were previously described^121^. After cross with *E2a-Cre*, genotyping was performed using specific primers to identify the germline delta allele. *Rax-Cre* mice were previously described^55^. Primers spanning the *Cre* and the *Rax* sequences were used for PCR genotyping. *Rax-Cre* mice were used to generate conditional mice (*Glut1*^flox/delta^; *Rax-Cre, Ldha*^flox /delta^; *Rax-Cre*); only one flox allele remains upon conditional Cre expression. Embryonic stages were assessed from the day of the vaginal plug that was designated as embryonic day 0.5 (E0.5). Strains are maintained in a mixed C57B6/NMRI background. The embryos were dissected in DMEM and observed under a microscope (Leica). All experimental procedures involving animals in this study were approved by the Institutional Animal Care and Use Committee and Northwestern University. Throughout the study, mice were housed at the Center for Comparative Medicine of Northwestern University (Chicago, IL, USA). Animals were maintained on a standard 12 h lighting cycle in a vivarium maintained at 21–23 °C, relative humidity of 30–70% and received unrestricted access to standard mouse diet (Teklad #7912, Envigo, Indianapolis, IN) and water.

### Derivation of conditional *Ldha* null ESCs

The conditional *Ldha* null and heterozygous ESCs were derived as described previously^41^. Briefly, irradiated mouse embryonic fibroblasts (MEFs) feeder cells were seeded prior to the isolation of blastocysts (E3.5). Isolated E3.5 embryos were transferred to MEFs in a conventional ESC maintenance medium^122^ supplemented with R2i (two chemical inhibitors, 10 μM SB431542 and 1 μM PD0325901). Typically, embryos were well-adhered to the culture dish with MEFs and hatched in 2 days. On day-7, dissociation into single cells was performed using TrypLE Express Enzyme when outgrowth of the inner cell mass (ICM) became evident. Cells were then continuously cultured in the ESC maintenance medium now supplemented with 2i (two chemical inhibitors, 3 μM CHIR99021 and 1 μM PD0325901) in the presence of MEFs. Optionally, the addition of Y-27632 (10 µM) for the first 2 days improved cell survival after single cell dissociation. Genotyping of each ESC line was performed after the complete removal of MEFs in a non-feeder culture. Primer sequences for genotyping: *Ldha flox* and wild-type alleles, Ldha-F 33676, 5’- CTGAGCACACCCATGTGAGA-3’; Ldha-R 33677, 5’- AGCAACACTCCAAGTCAGGA-3’; *Ldha delta* allele, Ldha-F 33676, 5’- CTGAGCACACCCATGTGAGA −3’; Ldha-R2 delta, 5’- AGTGTCTTCTCTTCCCTCAC −3’. Rax-Cre allele, Rax-Cre-F, 5’- AGATGCCAGGACATCAGGAACCTG-3’; Rax-Cre-R, 5’- ATCAGCCACACCAGACACAGAGATC −3’. Pluripotency of each clone was initially evaluated by alkaline phosphatase staining and immunostaining by combinations of embryonic stem cell markers. Cells were passaged every 3 days for ESC maintenance. For the eye organoid differentiation protocol, MEFs were removed from the 0.1% (wt/vol) gelatin-coated dish to isolate floating ESCs alone. The standard protocol was used to perform SFEBq (serum-free floating culture of embryoid body-like aggregates with quick aggregation) in Nunclon Sphera 96-Well, Nunclon Sphera-Treated, U-Shaped-Bottom Microplate (ThermoFisher Scientific, 174929 or 174925) on day 0. On day 1, Matrigel Matrix growth factor reduced (final concentration 100 μg/mL) (Corning, 354230) was added to the aggregates in retina differentiation medium^39^. Embryonic stem cell–qualified fetal bovine serum, KSR and Matrigel Matrix were used for the eye organoid culture.

### Glucose uptake imaging of mouse embryos

Imaging glucose uptake in mouse embryos was performed as described previously^21,22^. Briefly, freshly dissected E8.0 or E8.5 embryos were washed twice with a glucose blank medium (Thermo, A14430-01) and then incubated in RDM containing 2-NBDG at a final concentration of 0.5 mM (ThermoFisher Scientific, N13195) for 1 h (37’C and 60% O_2_, 5%CO_2)_. Embryos were washed with the medium 3 times for 30 min in total. Imaging was performed under a fluorescent microscope (EVOS) and analyzed by ImageJ (NIH). 2-NBDG uptake in the anterior neural plate was quantified relative to heart tissues.

### Immunohistochemistry

Standard protocols for embryo isolation^41,71^ were used. Briefly, embryos for immunohistochemistry were dissected in PBS and fixed in 4% paraformaldehyde in 0.1 M PBS (pH 7.4). After overnight protection in 30% sucrose/PBS, tissues were embedded in sucrose:OCT (Tissue-Tek) and sectioned in a cryostat. The following antibodies were used: anti-Rax (1:1000; TaKaRa, M229), anti-GFP (1:1000; Abcam, ab13970), Laminin (1:1000, Sigma-Aldrich, L9393), anti-Six3 (1:1000, custom made, Rockland), anti-Lhx5 (1:250, R&D systems, AF6290), anti-active Caspase-3 (1:500; BD Pharmingen, 559565), Cre (1:500; Chemicon, MAB3120, clone 2D8) and LDHA (1:500; Abcam, ab47010). The following secondary antibodies were used: Alexa 488–conjugated goat anti-chicken (1:1000, A11039, Invitrogen). Alexa 488–conjugated donkey anti-rabbit (1:1000, A-21206, Invitrogen), Alexa 488–conjugated donkey anti-goat (1:1000, A-11055, Invitrogen), Cy3-conjugated donkey anti-rabbit (1:200, 711-165-152, Jackson ImmunoResearch), Cy3-conjugated donkey anti-mouse (1:200, 715-165-151, Jackson ImmunoResearch), Cy3-conjugated donkey anti-goat (1:200, 705-165-147, Jackson ImmunoResearch), Cy5-conjugated donkey anti-goat (1:200, 705-495-147, Jackson ImmunoResearch), Jackson ImmunoResearch), Cy5-conjugated donkey anti-rabbit (1:200, 711-495-152, Jackson ImmunoResearch). CY5-conjugated goat anti-Guinea Pig (1:200, Jackson 106-175-003, Jackson ImmunoResearch). DAPI (1:1000) was used for DNA. Phalloidin was used to detect F-Actin. Images were obtained in a Zeiss Axioscope and analyzed by ImageJ (NIH).

### Quantitative PCR

As previously described^41^, the eye organoids were harvested at different days of differentiation and mRNA was isolated using RNeasy Mini Kit (QIAGEN). cDNA was generated (Clontech Laboratories) and 20 ng was used for qRT-PCR amplification using SYBR green. Primer sequences used in this study: *Gapdh*, forward, 5’-GGCATTGCTCTCAATGACAA-3’; reverse, 5’- CCCTGTTGCTGTAGCCGTAT-3’; *Six3*, forward, 5’-TCAGCAGAGTCACCGTCCAC-3’; reverse, 5’- TACCGAGAGGATCGAAGTGC-3’; *Rax*, forward, 5’-CGACGTTCACCACTTACCAA-3’; reverse, 5’- TCGGTTCTGGAACCATACCT-3’; *Pax6*, forward, 5’-CAAACAACCTGCCTATGCAA-3’; reverse, 5’- GGCAGCATGCACGAGTATGA-3’; *Lhx2*, forward, 5’- CAGCTTGCGCAAAAGACC −3’; reverse, 5’- TAAAAGGTTGCGCCTGAACT −3’. *Sox2*, forward, 5’- AACTTTTGTCCGAGACCGAGAA −3’; reverse, 5’- CCTCCGGGAAGCGTGTACT −3’; *Ldha*, forward, 5’- GGCACTGACGCAGACAAG −3’; reverse, 5’- TGATCACCTCGTAGGCACTG −3’. *Gapdh* was used as a housekeeping gene (i.e., its expression was used to normalize gene expression levels). The relative level of each gene was calculated using the 2^−ΔΔCt^ method and reported as relative fold-change mean ± S.E.M.

### Glucose and lactate isotope labeling

For glucose or lactate labeling^61,123^, glucose-free RPMI medium was supplemented with 10% dialyzed FBS and U^13^C-glucose (2 g/liter; Cambridge Isotope Laboratories) or U^13^C-lactate (2 g/liter; Cambridge Isotope Laboratories). All the organoids (50 organoids per condition) were washed with blank SILAC before being reconstituted in ^13^C medium at a concentration of 10 eye organoids/ml. The incubation was performed from 15 min to 2 h for time-course analysis of glucose flux. For the analysis in Fig. 4G and Supplementary Fig. 4, LDH inhibitor (GNE-140) treatment was done 30 min prior to the isotopic labeling experiments and the treatment continued until sample collection (^13^C labeling time was 30 min). The eye organoids were collected and washed twice with PBS before pellets were flash-frozen and stored at −80 °C until metabolite extraction. Metabolite analysis was performed as described previously^124^. Briefly, for metabolite extraction, 80% methanol was used followed by the rapid freeze-thaw method to break the tissues. The supernatant underwent speedvac drying. The samples were prepared in 80% acetonitrile and were analyzed by High-Resolution Liquid Chromatography Tandem Mass Spectrometry (LC-MS/MS). To control for the same concentration of protein/metabolites used as input for LC-MS/MS, we performed a series of controls as follows: 1. The same number of organoids are used for metabolite extraction; 2. When metabolite levels were analyzed, all data were normalized to total ion count/chromatogram (TIC)^125^; 3. When labeling data was analyzed, all isotopic enrichment for each metabolite were calculated against the total pool of unlabeled for each metabolite. For analyzing ^13^C-glucose labeling, the standard % enrichment (fractional enrichment displayed as percentages) was calculated using the normalized peak area of each metabolite’s isotopologue over its total pool as described in ref. ^126^. All data including TIC and unlabeled pools for each metabolite is available upon request.

### Extracellular acidification and oxygen consumption rate measurements

Extracellular acidification rate (ECAR) and oxygen consumption rate (OCR) were measured using an XF96 extracellular flux analyzer (Seahorse Bioscience) as previously described^124,127^. XF96 cell culture plates were coated with Cell-Tak (Corning, Cat. # 354240) to enhance immobilization of samples at the bottom. Typically, one eye organoid contains 10 thousand cells. The organoids were washed and resuspended with Seahorse XF base medium without phenol red (Agilent, Cat# 103335-100) supplemented with L-Glutamine (2 mM; Millipore Sigma, Cat. #G8540). Five to eight organoids per well were plated onto the XF96 plates and spun down at 400 × *g* for 2 min without deceleration. Prior to performing the assay, plates were incubated at 37’C without CO_2_. First, 10 mM glucose or lactate were injected to the seahorse microplates. After three initial readings, oligomycin (2 μM; Agilent, Cat. #103015-100) was injected into each well. Next, to monitor glycolytic activity, 2-DG (25 mM) or GNE-140 (20 µM) were added to the plate followed by three readings to complete ECAR and OCR tracing.

### RNA sequencing

The eye organoids were harvested (four biological independent experiments) and total RNA was isolated using RNeasy Mini Kit (QIAGEN). Prior to the library preparation, RNA quantification was performed by Qubit (Thermo Fisher). Next, a high sensitivity RNA assay was used to determine the quality of total RNA (bioanalyzer, Agilent Technologies). TruSeq mRNA-Seq Library Prep was performed, and the libraries were sequenced by Illumina HiSeq 4000 (50 bp, Single-Read, 300 million Reads) in the sequencing core at Northwestern University. Bioinformatic analysis generated Heatmap and Venn diagrams. The genes are ordered or sorted by log fold change to split them into upregulated and downregulated sets. The significant genes that had an FDR-adjusted *p*-value < 0.05 as thresholds/cutoffs were included in the Venn diagram generated using BioVenn^128^. The RNA-seq gene expression heatmaps were created with R version 3.3.1.

### ChIP sequencing

ChIP was performed as described previously^129^. Briefly, 100 eye organoids per experiment were harvested and crosslinked adding 1% formaldehyde/PBS in suspension for 20 min at RT on a rocker and quenched using 125 mM Glycine. The organoids were spun down and snap-freezed in liquid nitrogen. 300 organoids from three biologically independent experiments were pooled per treatment condition. This study performed two sets of ChIP sequencing from six biologically independent experiments. The samples were sonicated using a Covaris E220 system. Sonication conditions were 20% duty factor for histones, peak intensity pulse 140, and 200 cycles per burst for 4 min. Detergent compatible (DC) protein assay (Bio-Rad Laboratories) for protein quantification were performed to normalize each sample. Anti-Acetyl-Histone H3 (Lys27) (1:50, Cell Signaling technology, #8173S) was used for chromatin precipitation overnight on a rotator at 4’C. Chromatin immunoprecipitated DNA purification was done using ChIP DNA Clean & Concentrator (ZYMO RESEARCH, D5205). Prior to the library preparation, high sensitivity DNA assay was used to determine the quality of total DNA (bioanalyzer, Agilent Technologies). DNA-Seq library preparation was performed, and the libraries were sequenced by Illumina HiSeq 4000 (50 bp, Single-Read, 300 million Reads) in the sequencing core at Northwestern University. Bioinformatic analysis generated Annotated Peaks and Bigwig files. The command for Peak calling parameters using sample Kac_control as an example is: findPeaks Kac_control -style histone -o auto -i Input &> Kac_control_findPeaks.log. For data quality, Peaks at FDR 5% and above 5-fold enrichment were used as follows: H3K27ac_control (825105), H3K27ac_LDHi (1056232), H3K27ac_rescue (1036266). The data was visualized by a software, IGV_2.4.14 (Integrative Genomics Viewer, Broad Institute). The occupancy heatmaps (ChIP-seq) were created using deepTools version 2.5.4.

### Statistics & reproducibility

All experiments were independently performed at least 3 times except for the metabolomics analysis in Fig. 4B, as another time point (e.g., 60 min) shows a similar trend of isotopic labeling after it reaches a steady state. No data were excluded from the analyses. Representative micrographs are shown, as similar results were obtained from independent replicas. Values are expressed as mean ± S.E.M (standard error of the mean). Results were analyzed using GraphPad (GraphPad version 9.5.1) and Microsoft Excel. For statistical significance test, Student’s *t* test and one-way ANOVA were used for two-group and multiple-group comparison, respectively. In addition, post-hoc Tukey’s (all groups) or Dunnett’s test (versus control) was applied to compare multiple groups. Differences with *p*-value < 0.05 were considered statistically significant. *, ** or *** indicates a *p*-value is less than 0.05, 0.01 or 0.001, respectively. ns non-significance.

### Reporting summary

Further information on research design is available in the Nature Portfolio Reporting Summary linked to this article.

## Supplementary information

Supplementary Information

Reporting Summary

## Data Availability

The materials used in this study are available upon request. The scRNAseq, RNAseq and ChIPseq omics data is compiled under GEO accession number GSE202759. The metabolomics data in this study is available at the NIH Common Fund’s National Metabolomics Data Repository (NMDR) website, the Metabolomics Workbench, https://www.metabolomicsworkbench.org where it has been assigned Project ID PR001619. The data can be accessed directly via it’s Project 10.21228/M8F41P. Source data are provided with this paper.

## Source data

Source Data
