# Supplementary material for: Lactate-dependent transcriptional regulation controls mammalian eye morphogenesis
Source: Nat Commun. 2023 Jul 14;14:4129. doi: 10.1038/s41467-023-39672-2 (PMC10349100; doi:10.1038/s41467-023-39672-2)
Supplement: Supplementary file 1 — Supplementary Information [file 41467_2023_39672_MOESM1_ESM.pdf]

Supplementary Information for

Lactate-dependent Transcriptional Regulation Controls Mammalian Eye Morphogenesis

Authors: Nozomu Takata, Jason M. Miska, Marc A. Morgan, Priyam Patel, Leah K. Billingham, Neha Joshi, Matthew J. Schipma, Zachary J. Dumar, Nikita R. Joshi, Alexander V. Misharin, Ryan B. Embry, Luciano Fiore, Peng Gao, Lauren P. Diebold, Gregory S. McElroy, Ali Shilatifard, Navdeep S. Chandel, and Guillermo Oliver

Supplementary Figure 1. Characterization of glycolysis inhibitors on cell death and stage specificity

Supplementary Figure 2. Natural abundance of isotopic metabolites in eye organoids

Supplementary Figure 3. Pyruvate transport to mitochondria was not required for eye gene expression

Supplementary Figure 4. Pentose pathway is not required during eye morphogenesis

Supplementary Figure 5. Medium acidification or membrane permeable pyruvate cannot rescue the eye phenotype caused by LDHi

Supplementary Figure 6. Generation and characterization of conditional *Ldha* null mutant organoids

Supplementary Figure 7. Local change in H3K27ac following treatment with GNE-140 and lactate

Supplementary Figure 8. Depletion of Histone acetyltransferase (CBP/p300) activity reduced eye gene expression

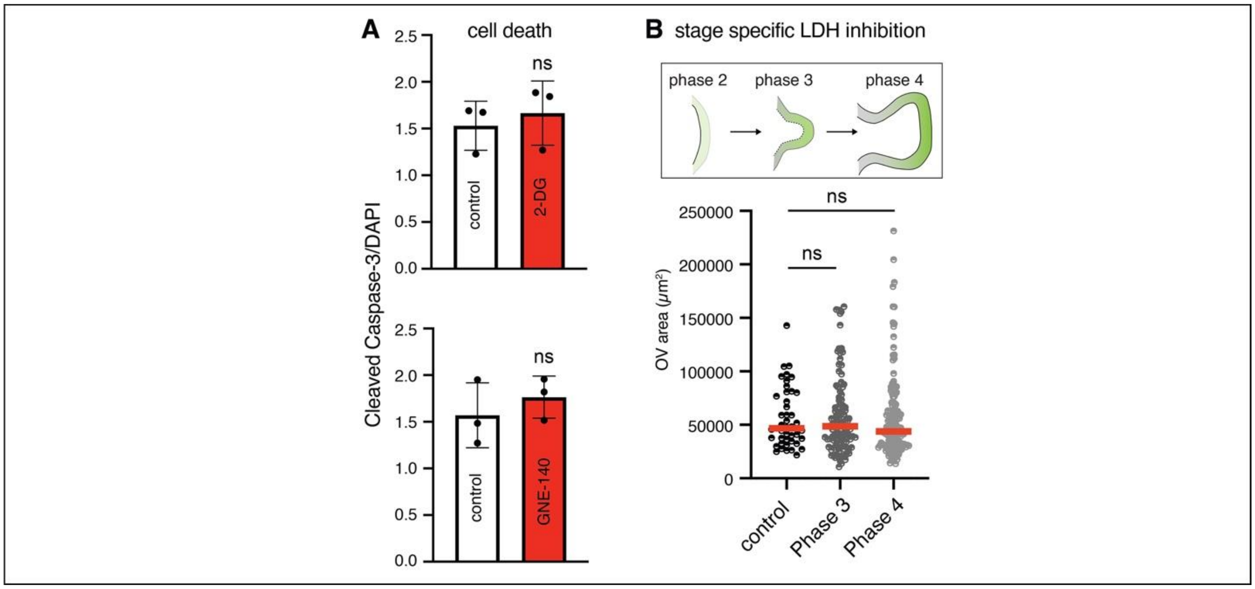

Supplementary Figure 1 | Characterization of glycolysis inhibitors on cell death and stage specificity**.** (**A**) Cleaved caspase-3 staining was done on cryosections in the presence of 2-DG (2 mM) or GNE-140, LDH inhibitors (20 µM). The fluorescence signals are measured by ImageJ. Cell death is calculated by the Cleaved caspase-3 signal intensity over that of DAPI as internal staining control. ns = Not significant. (**B**) Progress in eye morphogenesis is evaluated by the optic vesicle (OV) area at each time-point via ImageJ. The late-stage addition of LDHi did not show any significant impact on eye morphogenesis. Unpaired Student *t*-test (two-tailed) was performed (A, B). n.s. not significant. (A, B). Data are presented as mean values +/- SEM. Source data are provided as a Source Data file. n=3 biologically independent experiments were performed.

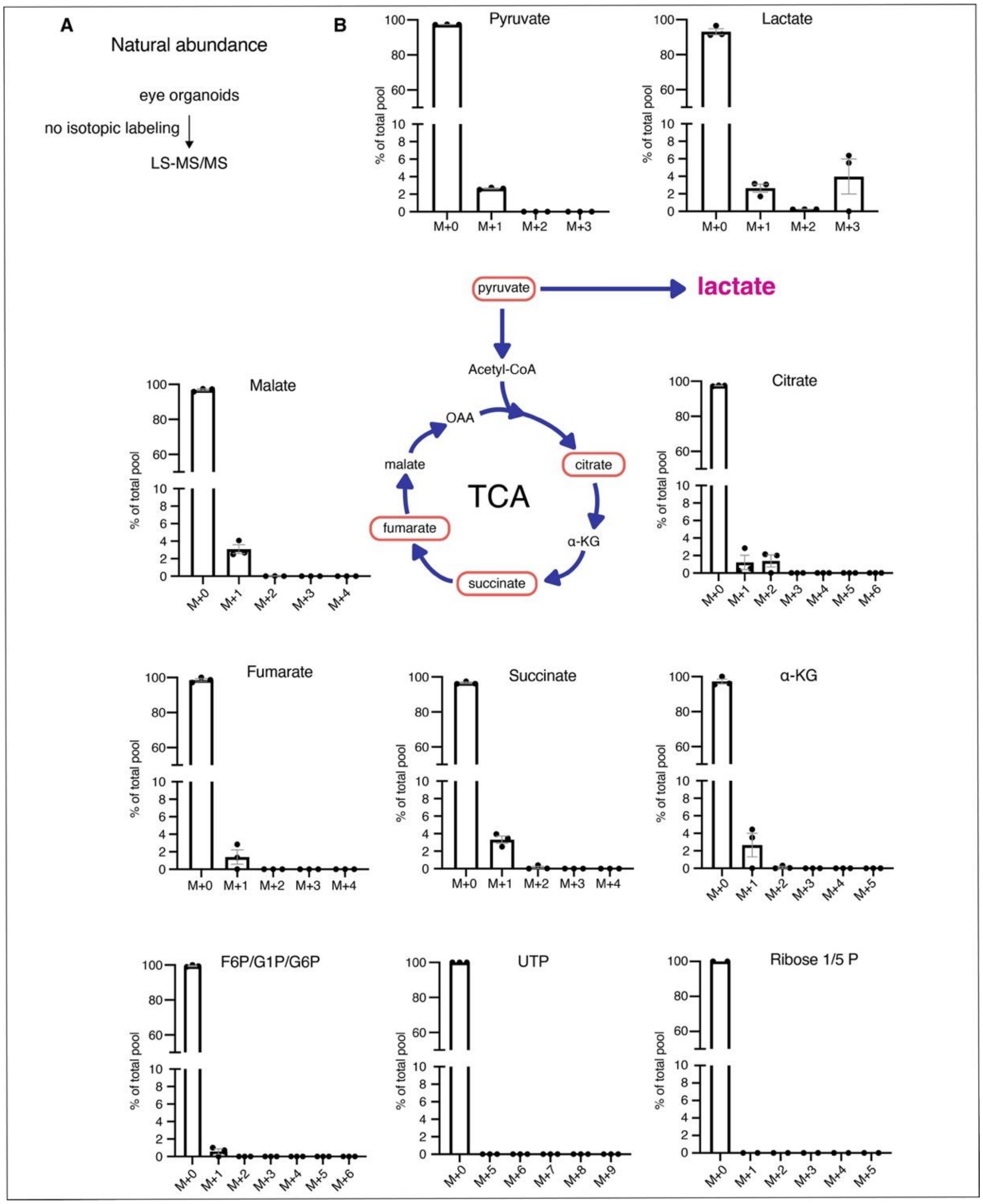

Supplementary Figure 2 | Natural abundance of isotopic metabolites in eye organoids. (**A, B**) Natural abundance was analyzed by LC-MS/MS without addition of ^{13}C-glucose to the eye organoid culture. All the metabolites detected show a negligible amount of naturally occurring isotopic signals below 3%, except lactate which was up to 5%. Data are presented as mean values +/- SEM (H). Source data are provided as a Source Data file. n=3 biologically independent experiments were performed.

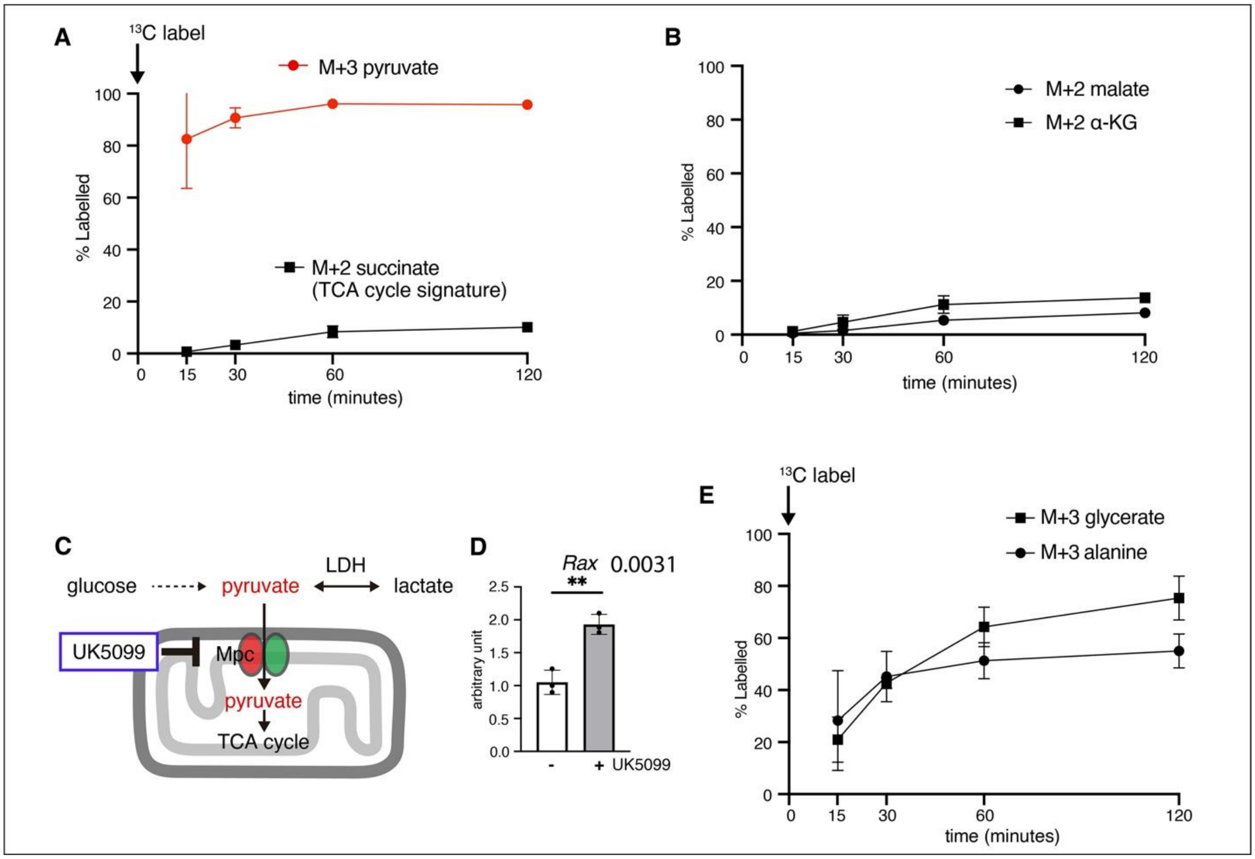

Supplementary Figure 3 | Pyruvate transport to mitochondria is not required for eye gene expression. (**A, B**) The direct quantification of glucose consumption with ^{13}C glucose tracing show rapid accumulation of pyruvate, while the levels of the TCA cycle signature succinate are extremely low. In addition, other TCA cycle metabolites such as Malate and α-Ketoglutarate (α-KG) are also lower. (**C, D**) The addition of 1 µM UK5099, an inhibitor against the mitochondrial pyruvate carrier (MPC) do not show any dramatic effect on *Rax* expression. (**E**) ^{13}C-labeled alanine and glycerate are detected in noticeable amounts. Unpaired Student *t*-test (two-tailed) was performed (D). ** indicates a p-value is less than 0.01. Data are presented as mean values +/- SEM (D). Source data are provided as a Source Data file. n=3 biologically independent experiments were performed.

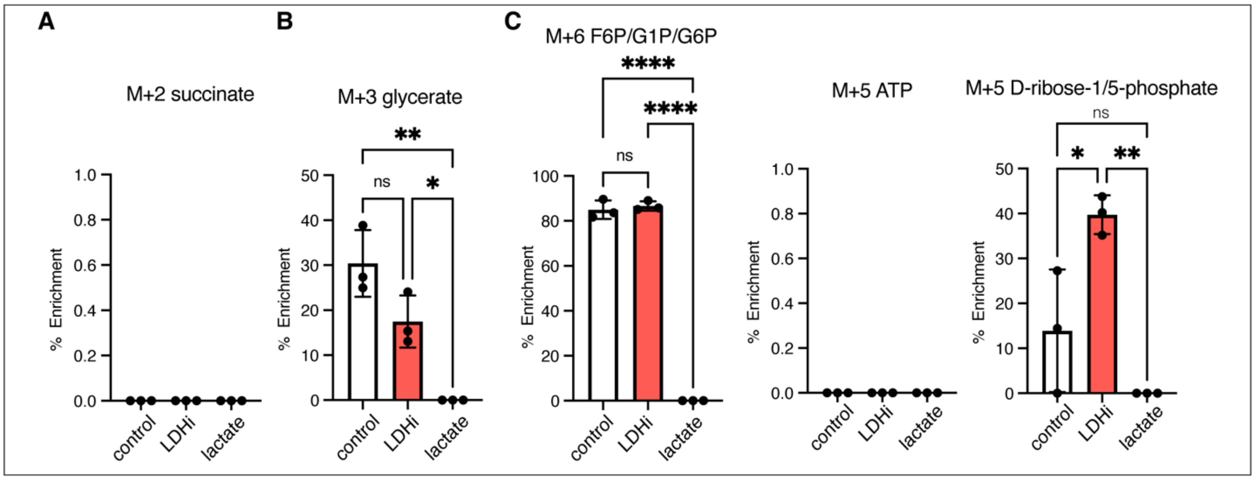

Supplementary Figure 4 | Pentose pathway is not required during eye morphogenesis. (**A-C**) Levels of succinate, glycerate, F6P/G1P/G6P, UTP and D-ribose were analyzed by ^{13}C isotopic labeling in the presence of the LDH inhibitor (20 µM); however, those metabolites show no significant differences regardless of LDH activity. One-way ANOVA followed by Tukey's post-hoc test was performed (B, C). ****$p < 0.0001$, **$p < 0.01$, *$p < 0.05$, n.s. not significant. Data are presented as mean values +/- SEM (B, C). Source data are provided as a Source Data file. n=3 biologically independent experiments were performed.

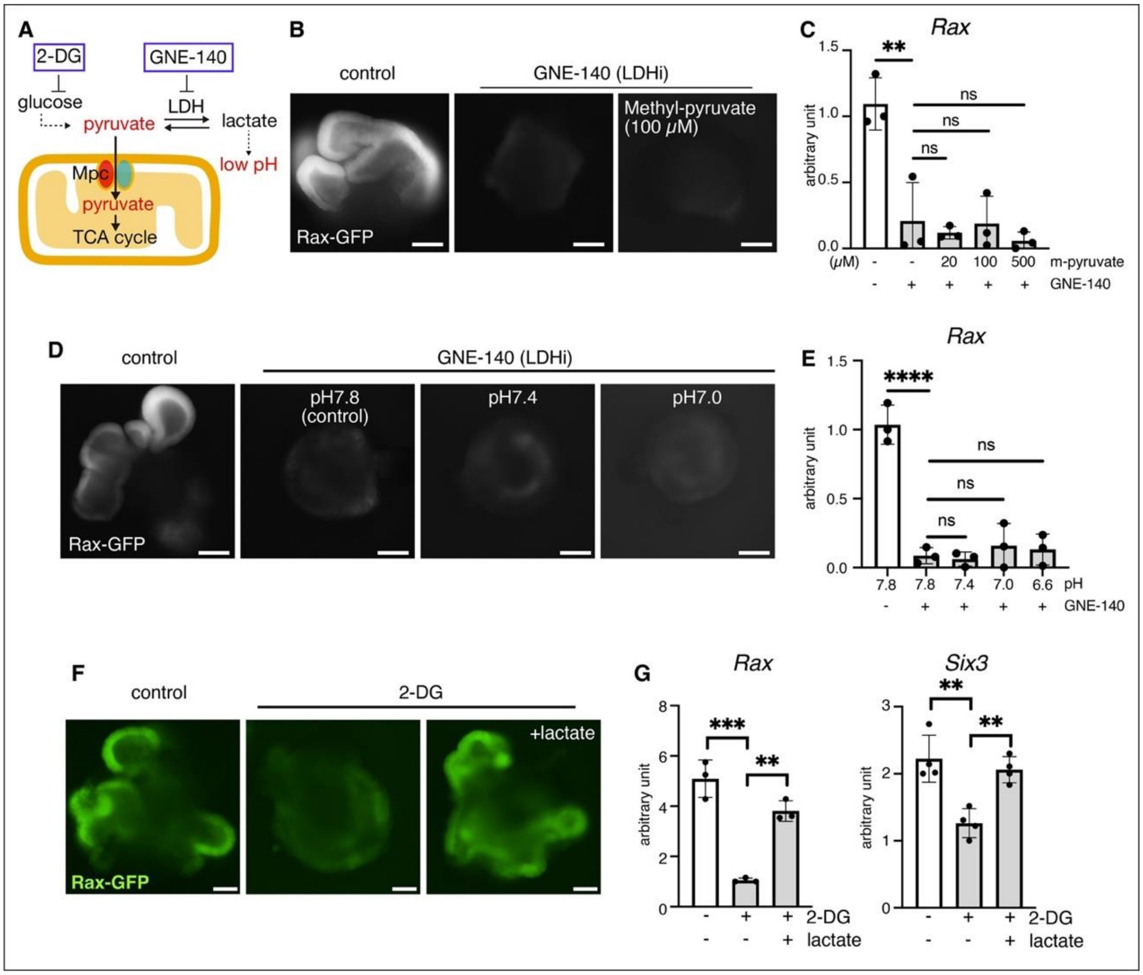

Supplementary Figure 5 | Medium acidification or membrane permeable pyruvate cannot rescue the eye phenotype caused by LDHi. (**A-C**) lactate can reverse back to pyruvate to be utilized by mitochondria; however, pyruvate do not rescue the phenotype caused by LDHi (20 µM). M-pyruvate = methyl-pyruvate. (**D, E**) During production of endogenous lactate, intercellular pH gets reduced, but lowering pH in the culture medium do not affect eye gene expression or eye morphogenesis. Those two tests suggest that pyruvate used in mitochondria and low pH are unlikely major participants in eye morphogenesis. (**F**) The addition of sodium L-lactate (25 mM) which does not change the pH, significantly rescued the OV phenotype in the presence of the glucose analogue 2-DG (2 mM). (**G**) Quantification analysis using RT-qPCR confirm that *Rax* and *Six3* expression was rescued by the addition of sodium L-lactate. Scale bar, 100 µm (B, D, F). One-way ANOVA followed by Tukey's post-hoc test was performed (C, E, G). ****$p < 0.0001$, ***$p < 0.001$, **$p < 0.01$, n.s. not significant. Data are presented as mean values

+/- SEM (C, E, G). Source data are provided as a Source Data file. n=3 biologically independent experiments were performed.

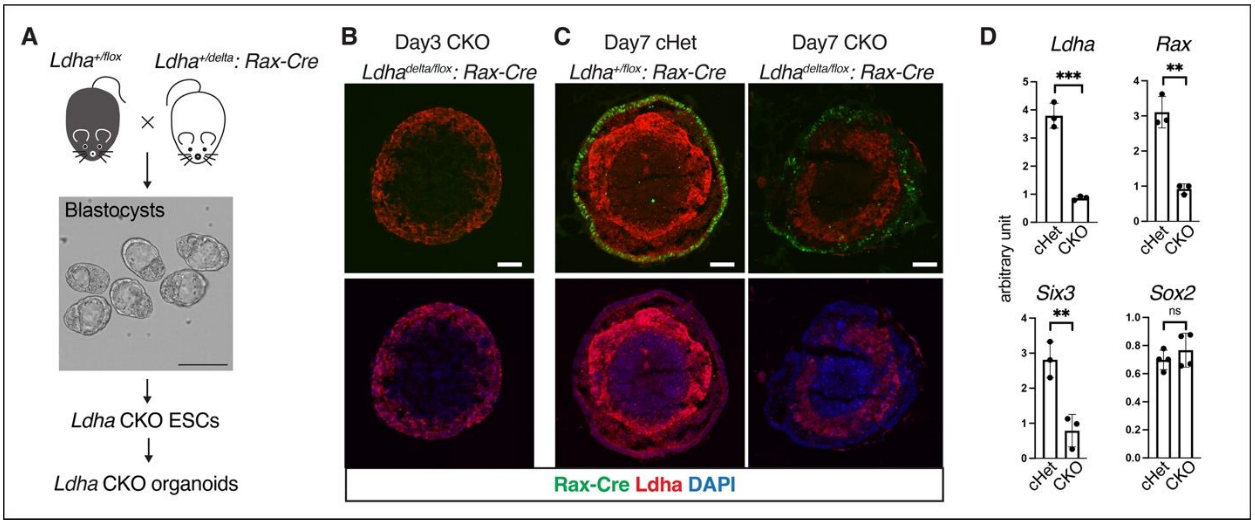

Supplementary Figure 6 | Generation and characterization of conditional LDHA null mutant organoids. (**A**) Isolation of blastocysts from the conditional mutant embryos. (**B, C**) Day3-CKO and day-7 Het organoids express Ldha, whereas day-7 CKO organoids show ablation of Ldha in cells positive for Rax-Cre. (**D**) Quantification of marker expression via RT-qPCR. In the mutant organoids, expression of the developmental eye markers *Rax* and *Six3* significantly reduced, while that of the pan-neural marker *Sox2* does not change. CKO=conditional knockout. cHet=conditional heterozygote. Scale bar, 100 µm (A-C). Unpaired Student *t*-test (two-tailed) was performed (D). ***$p < 0.001$, **$p < 0.01$, n.s. not significant. Data are presented as mean values +/- SEM (D). Source data are provided as a Source Data file. n=3 biologically independent experiments were performed.

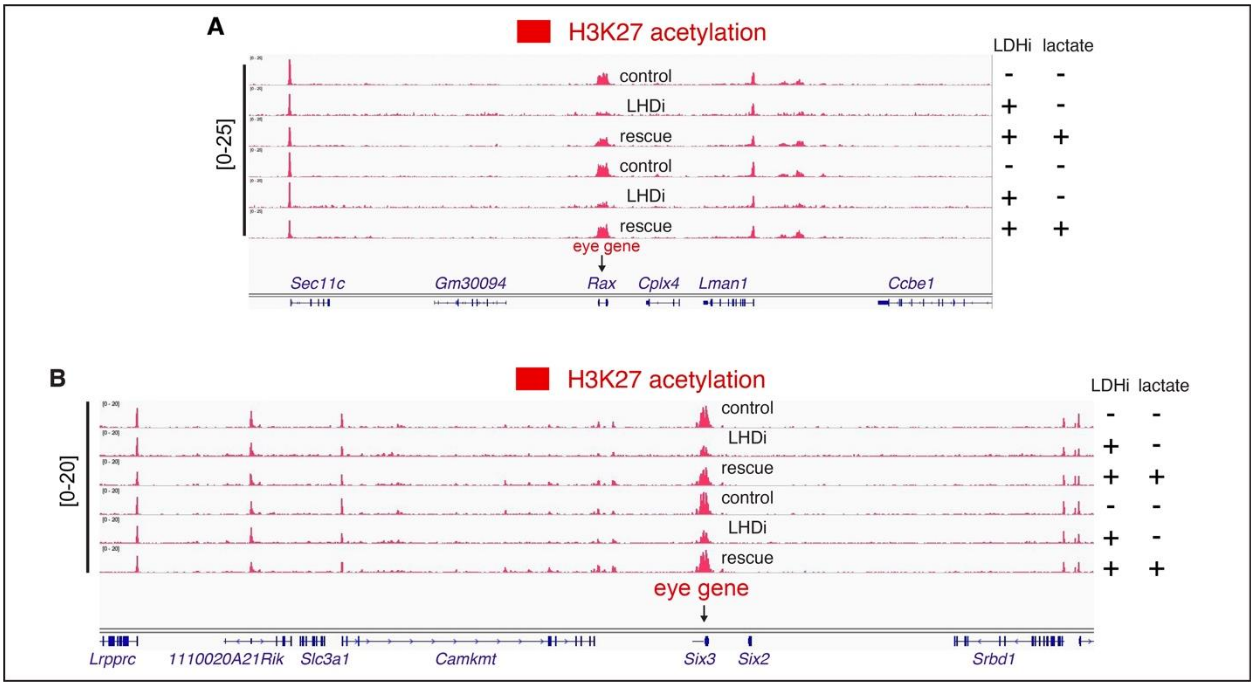

Supplementary Figure 7 | Local change in H3K27ac following treatment with GNE-140 and lactate. (A, B) H3K27ac peaks around the *Rax* and *Six3* loci showing the specificity of acetylation on eye developmental genes, as other genes are not affected by the deficiency in LDH activity nor lactate addition.

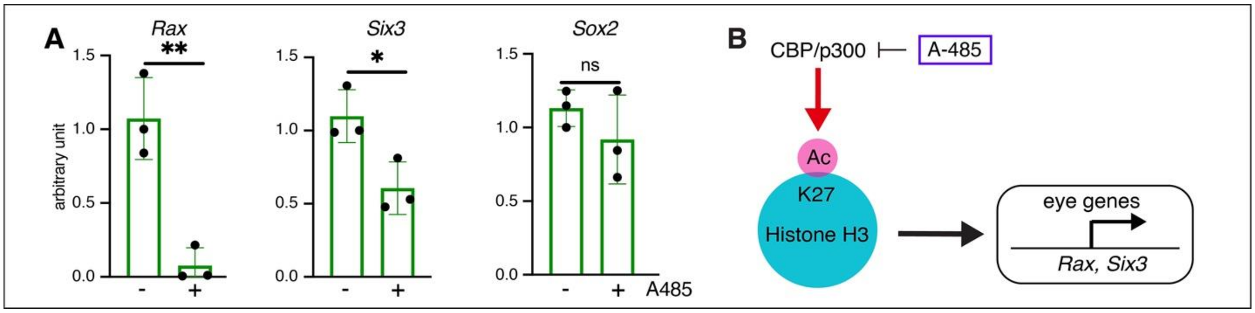

Supplementary Figure 8 | Depletion of Histone acetyltransferase (CBP/p300) activity reduces eye gene expression. (**A**) A-485 is a potent Histone acetyltransferase (HAT) inhibitor. Quantification analysis using RT-qPCR shows that *Rax* and *Six3* expression in day 5 cultures are significantly reduced by the addition of A-485 (2 µM) from day 4. Pan-neural marker *Sox2* does not show significant change. (**B**) Schematic diagram showing CBP/p300 mediated H3K27 acetylation. Unpaired Student *t*-test (two-tailed) was performed (A). $^{**}p < 0.01$, $^{*}p < 0.05$, n.s. not significant (A). Data are presented as mean values +/- SEM (A). Source data are provided as a Source Data file. n=3 biologically independent experiments were performed.
