# Supplementary material for: Lactate-dependent transcriptional regulation controls mammalian eye morphogenesis
Source: Nat Commun. 2023 Jul 14;14:4129. doi: 10.1038/s41467-023-39672-2 (PMC10349100; doi:10.1038/s41467-023-39672-2)
Supplement: Supplementary file 2 — Reporting Summary [file 41467_2023_39672_MOESM2_ESM.pdf]

nature portfolio

Corresponding author(s): *Double-blind peer review submissions: write DBPR and your manuscript number here instead of author names.*

Last updated by author(s): *YYYY-MM-DD*

Reporting Summary

Nature Portfolio wishes to improve the reproducibility of the work that we publish. This form provides structure for consistency and transparency in reporting. For further information on Nature Portfolio policies, see our Editorial Policies and the Editorial Policy Checklist.

Statistics

For all statistical analyses, confirm that the following items are present in the figure legend, table legend, main text, or Methods section.

n/a	Confirmed	
☐	☒	The exact sample size (*n*) for each experimental group/condition, given as a discrete number and unit of measurement
☐	☒	A statement on whether measurements were taken from distinct samples or whether the same sample was measured repeatedly
☐	☒	The statistical test(s) used AND whether they are one- or two-sided<br>*Only common tests should be described solely by name; describe more complex techniques in the Methods section.*
☐	☒	A description of all covariates tested
☐	☒	A description of any assumptions or corrections, such as tests of normality and adjustment for multiple comparisons
☐	☒	A full description of the statistical parameters including central tendency (e.g. means) or other basic estimates (e.g. regression coefficient) AND variation (e.g. standard deviation) or associated estimates of uncertainty (e.g. confidence intervals)
☐	☒	For null hypothesis testing, the test statistic (e.g. *F*, *t*, *r*) with confidence intervals, effect sizes, degrees of freedom and *P* value noted<br>*Give P values as exact values whenever suitable.*
☒	☐	For Bayesian analysis, information on the choice of priors and Markov chain Monte Carlo settings
☒	☐	For hierarchical and complex designs, identification of the appropriate level for tests and full reporting of outcomes
☒	☐	Estimates of effect sizes (e.g. Cohen's *d*, Pearson's *r*), indicating how they were calculated

Our web collection on statistics for biologists contains articles on many of the points above.

Software and code

Policy information about availability of computer code

Data collection	EVOS, Cell Imaging system, Zeiss Axioscope, Loupe Cell Browser 1.0.5, TapeStation 4200, Bioanalyzer, Integrative Genomics Viewer IGV_2.4.14., Quantstudio3, XF96 extracellular flux analyzer
Data analysis	GraphPad software 9.5.8, ImageJ, Microsoft Excel, Cell Ranger 7.0.0, Seurat v4.1.1, ScaleData, R version 3.3.1, BioVenn, deepTools version 2.5.4.

For manuscripts utilizing custom algorithms or software that are central to the research but not yet described in published literature, software must be made available to editors and reviewers. We strongly encourage code deposition in a community repository (e.g. GitHub). See the Nature Portfolio guidelines for submitting code & software for further information.

Data

Policy information about availability of data

All manuscripts must include a data availability statement. This statement should provide the following information, where applicable:

- Accession codes, unique identifiers, or web links for publicly available datasets
- A description of any restrictions on data availability
- For clinical datasets or third party data, please ensure that the statement adheres to our policy

The omics data is compiled under GEO accession number is (GSE202759). Metabolomics Workbench, https://www.metabolomicsworkbench.org where it has been assigned Project ID PR001619. The data can be accessed directly via it's Project DOI: https://doi.org/10.21228/M8F41P. This work is supported by Metabolomics Workbench/National Metabolomics Data Repository (NMDR) (grant# U2C-DK119886), Common Fund Data Ecosystem (CFDE) (grant# 3OT2OD030544) and

Metabolomics Consortium Coordinating Center (M3C) (grant# 1U2C-DK119889). All data including isotopically labeled samples and unlabeled pools for each metabolite is available upon request.

Human research participants

Policy information about studies involving human research participants and Sex and Gender in Research.

Reporting on sex and gender	Not applicable.
Population characteristics	Not applicable.
Recruitment	Not applicable.
Ethics oversight	Not applicable.

Note that full information on the approval of the study protocol must also be provided in the manuscript.

Field-specific reporting

Please select the one below that is the best fit for your research. If you are not sure, read the appropriate sections before making your selection.

☒ Life sciences ☐ Behavioural & social sciences ☐ Ecological, evolutionary & environmental sciences

For a reference copy of the document with all sections, see nature.com/documents/nr-reporting-summary-flat.pdf

Life sciences study design

All studies must disclose on these points even when the disclosure is negative.

Sample size	Sample size was determined based on standard protocols in the fields. Three biological triplicates were performed.
Data exclusions	If endogenous genes or proteins were not eliminated clearly in the conditional mutant embryos, they were excluded for further analysis.
Replication	All experiments were done multiple times independently to ensure data reproducibility, and stated in the manuscript.
Randomization	Since genotyping determined the embryos to be further analyzed, no particular randomization was applied.
Blinding	Blind test was applied only in the glucose tracing assay as it requires live embryo imaging and then genotyping to identify the mutants and wild-type animals. None of the other experiments were blind because the experiments were designed to be grouped and treated based on the aims.

Reporting for specific materials, systems and methods

We require information from authors about some types of materials, experimental systems and methods used in many studies. Here, indicate whether each material, system or method listed is relevant to your study. If you are not sure if a list item applies to your research, read the appropriate section before selecting a response.

Materials & experimental systems

n/a	Involved in the study	
☐	☒	Antibodies
☐	☒	Eukaryotic cell lines
☒	☐	Palaeontology and archaeology
☐	☒	Animals and other organisms
☒	☐	Clinical data
☒	☐	Dual use research of concern

Methods

n/a	Involved in the study	
☐	☒	ChIP-seq
☒	☐	Flow cytometry
☒	☐	MRI-based neuroimaging

Antibodies

Antibodies used	anti-Acetyl-Histone H3 (Lys27) (1:50, Cell Signaling technology, #8173S), anti-Rax (1:1000; TaKaRa, M229), anti-GFP (1:1000; Abcam, ab13970), Laminin (1:1000, Sigma-Aldrich, L9393), anti-Six3 (1:1000, custom made, Rockland), anti-Lhx5 (1:250, R&D systems, AF6290), anti-active Caspase-3 (1:500; BD Pharmingen, 559565), Cre (1:500; Chemicon, MAB3120, clone 2D8) and LDHA (1:500; Abcam, ab47010). The following secondary antibodies were used: Alexa 488–conjugated goat anti-chicken (1:1000, A11039,

	Invitrogen). Alexa 488–conjugated donkey anti-rabbit (1:1000, A-21206, Invitrogen), Alexa 488–conjugated donkey anti-goat (1:1000, A-11055, Invitrogen), Cy3-conjugated donkey anti-rabbit (1:200, 711-165-152, Jackson ImmunoResearch), Cy3-conjugated donkey anti-mouse (1:200, 715-165-151, Jackson ImmunoResearch), Cy3-conjugated donkey anti-goat (1:200, 705-165-147, Jackson ImmunoResearch), Cy5-conjugated donkey anti-goat (1:200, 705-495-147, Jackson ImmunoResearch), Jackson ImmunoResearch), Cy5-conjugated donkey anti-rabbit (1:200, 711-495-152, Jackson ImmunoResearch). CY5-conjugated goat anti-Guinea Pig (1:200, Jackson 106-175-003, Jackson ImmunoResearch)
Validation	The antibodies were validated by immunostaining with mutant embryos or cells/tissues treated with agonists or antagonists. Besides, companies that provide those antibodies show their test results.

Eukaryotic cell lines

Policy information about cell lines and Sex and Gender in Research

Cell line source(s)	Mouse embryonic stem cell line Rax-EGFP was obtained originally from Yoshiki Sasai and now the line is deposited under the name AES0145: Rx-GFP K/I EB5 at RIKEN CELL BANK.
Authentication	The stem cell identity was regulary tested by Alkaline phosphatase asssay, immunostaining with Nanog, Oct3/4 and E-Cadherin.
Mycoplasma contamination	Rax-EGFP cells were tested negative for mycoplasma contamination.
Commonly misidentified lines (See ICLAC register)	Not applicable in this study.

Animals and other research organisms

Policy information about studies involving animals; ARRIVE guidelines recommended for reporting animal research, and Sex and Gender in Research

Laboratory animals	Six3LacZ, Rax KO, LDHA flox, Glut1 flox, Rax-Cre and E2a-Cre mice were maintained in a mixed C57B6 and NMRI background. Males and females from 12 weeks old to 6 months old were maintained and used for breeding and cross for dissecting embryos,
Wild animals	Not applicable in this study.
Reporting on sex	Animal study was not designed to select sex for the analysis. Both male and female animals should be equally included.
Field-collected samples	Not applicable in this study.
Ethics oversight	All animal husbandry was performed in accordance with protocols approved by the Institutional Animal Care and Use Committee and Northwestern University.

Note that full information on the approval of the study protocol must also be provided in the manuscript.

ChIP-seq

Data deposition

- [x] Confirm that both raw and final processed data have been deposited in a public database such as GEO.
- [x] Confirm that you have deposited or provided access to graph files (e.g. BED files) for the called peaks.

Data access links *May remain private before publication.*	GSE202759
Files in database submission	The raw data files were provided upon submission.
Genome browser session (e.g. UCSC)	no longer applicable

Methodology

Replicates	This study performed two sets of ChIP sequencing from six biologically independent experiments in total.
Sequencing depth	50 bp, single-read, 300 million reads.
Antibodies	anti-Acetyl-Histone H3 (Lys27) (Cell Signaling technology, #8173S)
Peak calling parameters	The quality of reads, in FASTQ format, was evaluated using FastQC (version 0.11.7). FastQ Screen (version 0.14.0) was applied to the reads to ensure that they aligned to the appropriate reference genome. Reads were trimmed to remove Illumina adapters from the 3' ends using cutadapt (version 1.14). Trimmed reads were aligned to the Mus musculus genome (GRCm38 Ensembl release 102) using using Bowtie2 (version 2.2.9) with default parameters while only reads that mapped uniquely to the genome were used in subsequent analysis. Peak calling and the creation of UCSC read density tracks were performed using Hypergeometric Optimization

	of Motif EnRichment (HOMER, version 4.11) using default parameters and input samples to control for non-specific binding.
Data quality	We used the peak calling above and ensure data based on FDR 5% and above 5-fold enrichment. Below is Peaks at FDR 5% and above 5-fold enrichment: H3K27ac_control (825105), H3K27ac_LDHi (1056232), H3K27ac_rescue (1036266).
Software	Integrative Genomics Viewer IGV_2.4.14
